# Supplementary material for: The potential use of therapeutics and prophylactic mRNA vaccines in human papillomavirus (HPV)
Source: Virol J. 2024 May 31;21:124. doi: 10.1186/s12985-024-02397-9 (PMC11143593; doi:10.1186/s12985-024-02397-9)
Supplement: Supplementary file 1 — Supplementary Material 1. [file 12985_2024_2397_MOESM1_ESM.docx]

**The potential use of therapeutics and prophylactic mRNA vaccines in Human papillomavirus (HPV)**

**Fatemeh Movahed^1^, Satinik Darzi^2^, Parya Mahdavi^3^, Morug Salih Mahdi^4^, Omer Qutaiba B. Allela^5^, Hayder Naji Sameer^6^, Mohaned Adil^7^, Hasna Zarkhah^8*^, Saman Yasamineh^9*^, Omid Gholizadeh^10^**

1- Department of Gynecology and Obstetrics, School of Medicine, Tehran University of Medical Sciences, Tehran, Iran.

2- Department Of Obstetrics and Gynecology, Abnormal Uterine Bleeding Research Center, Semnan University of Medical Sciences, Semnan, Iran

3- Department of Obstetrics and Gynecology, Faculty of Medicine, Kermanshah University of Medical Sciences, Kermanshah, Iran.

4- College of MLT, Ahl Al Bayt University, Iraq

5- Department of Pharmacy, Al-Noor University College, Nineveh, Iraq

6- Collage of Pharmacy, National University of Science and Technology, Dhi Qar, 64001, Iraq

7- Pharmacy college, Al-Farahidi University, Iraq

8- Department of Obstetrics and Gynaecology, Tabriz University of Medical Siences, Tabriz, Iran

9- Young Researchers and Elite Club, Tabriz Branch, Islamic Azad University, Tabriz, Iran

10- Azad Researchers, Virology and Biotechnology, Tehran, Iran

**Corresponding author:**

**Hasna Zarkhah,** Department of Obstetrics and Gynaecology, Tabriz University of Medical Siences, Tabriz, Iran

Email: [hasnazarkhah@gmail.com](mailto:hasnazarkhah@gmail.com)

Tel: 09149127110

**Saman Yasamineh**, Young Researchers and Elite Club, Tabriz Branch, Islamic Azad University, Tabriz, Iran

**Tel:** +989140446754

**Email:** Yassaman124@gmail.com

**Abstract**

Cervical cancer (CC) and other malignant malignancies are acknowledged to be primarily caused by persistent human papillomavirus (HPV) infection. Historically, vaccinations against viruses that produce neutralizing antibodies unique to the virus have been an affordable way to manage viral diseases. CC risk is decreased, but not eliminated, by HPV vaccinations. Since vaccinations have been made available globally, almost 90% of HPV infections have been successfully avoided. On the lesions and diseases that are already present, however, no discernible treatment benefit has been shown. As a result, therapeutic vaccines that elicit immune responses mediated by cells are necessary for the treatment of established infections and cancers. mRNA vaccines possess remarkable potential in combating viral diseases and malignancy as a result of their superior industrial production, safety, and efficacy. Furthermore, considering the expeditiousness of production, the mRNA vaccine exhibits promise as a therapeutic approach targeting HPV. Given that the HPV-encoded early proteins, including oncoproteins E6 and E7, are consistently present in HPV-related cancers and pre-cancerous lesions and have crucial functions in the progression and persistence of HPV-related diseases, they serve as ideal targets for therapeutic HPV vaccines. The action mechanism of HPV and HPV-related cancer mRNA vaccines, their recent advancements in clinical trials, and the potential for their therapeutic applications are highlighted in this study, which also offers a quick summary of the present state of mRNA vaccines. Lastly, we highlight a few difficulties with mRNA HPV vaccination clinical practice and provide our thoughts on further advancements in this quickly changing sector. It is expected that mRNA vaccines will soon be produced quickly for clinical HPV prevention and treatment.

**Keywords: mRNA vaccines, Papillomavirus (HPV), Therapeutic, Prophylactic**

**
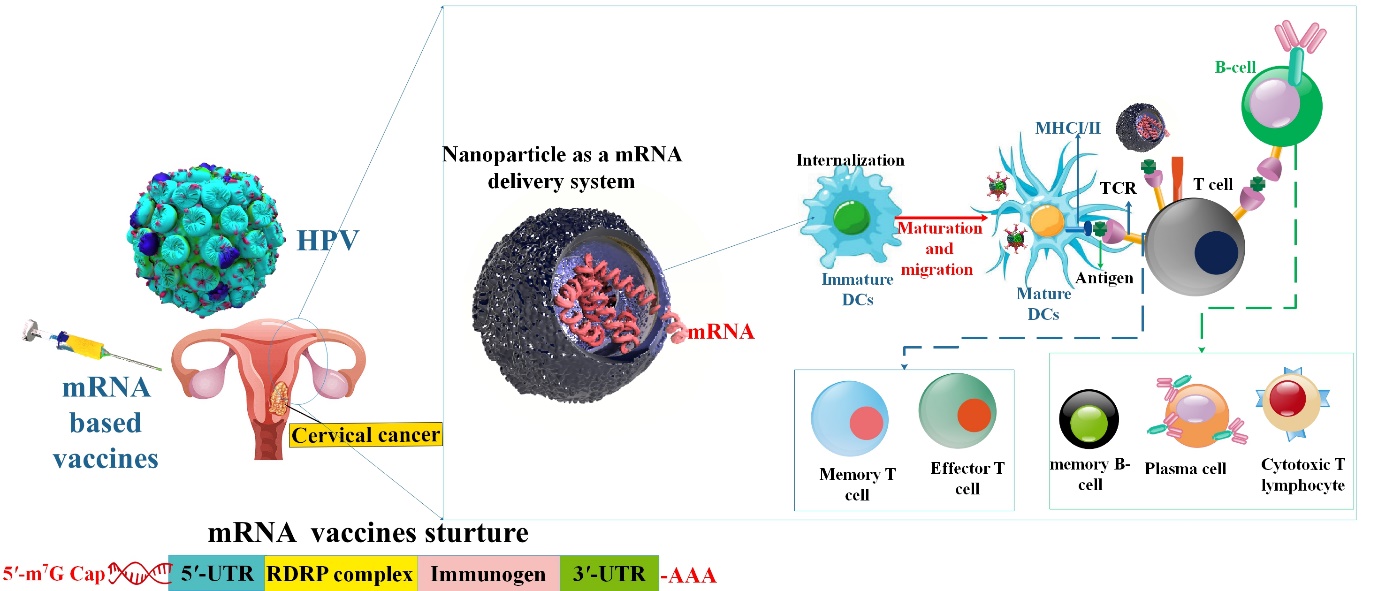
**

**1. Introduction**

Papillomaviruses are non-enveloped, icosahedral, 55 nm-diameter microorganisms with a circular, double-stranded (ds) DNA genome measuring approximately 8000 base pairs in length [1]. The majority of higher vertebrates, including birds and mammals, contain them, and they cause tumors to form on the skin and in the mucous membranes [2]. More than one hundred unique genotypes of human papillomavirus (HPV) are unique to the human species and the squamous stratified epithelium. Their malignant (squamous cell carcinomas) and benign (warts, papillomas, condylomas) tumors are opposite ends of the spectrum [3]. HPVs are a prodigious and time-honored assemblage of viruses that have evolved in tandem with their hosts to replicate within distinct anatomical compartments of the stratified epithelia [4]. The pathogens exhibit persistent replication in dividing cells, manipulate the cellular environment by hijacking critical host cellular processes, evade immune detection by producing virions in terminally differentiated cells that are ejected from the host, and do so in dividing cells [5].

Nevertheless, the majority of HPVs induce latent and asymptomatic infections unless the host's immune system is compromised. As of this moment, nearly 450 unique HPV strains' genomes have been isolated and sequenced [6]. Over 90% of instances of cervical cancer (CC) are linked to HPV. Multiple factors, such as geographical variation, HPV genotype, the specific population being studied, HPV vaccination status, and the location from which anatomical samples are collected, all have an impact on the occurrence and underlying mechanisms of HPV-induced cancer [7, 8]. Furthermore, preventive measures and vaccination awareness campaigns may contribute to a decline in the incidence and prevalence of HPV infection [9]. HPV-associated malignancies account for more than half of all head and neck carcinoma cases and nearly all cases of CC in women [10].

Several safe and efficacious vaccines, including the bivalent Cervarix^TM^ manufactured by GlaxoSmithKline and the quadrivalent Gardasil^TM^ and nonavalent Gardasil9, both manufactured by Merck, have obtained global licenses. Despite this, the inadequate infrastructure of fragile health systems in low and middle-income nations (LMICs) renders the administration of dual doses of the vaccine to adolescent females a formidable challenge [13, 14]. Vaccines represent the most economically viable approach to avert this worldwide dissemination and mitigate acute and chronic infections [15]. Developed in the last few decades, viral vector and nucleic acid-based vaccine platforms hold the potential to offer resolutions to the vaccine mentioned above challenges [16]. Nucleic acid-based vaccines supplied by non-viral vectors may be used to imitate infection or vaccination with living microbes [17]. [13, 14]

mRNA technology has the potential to revolutionize the conventional method of vaccine development [18]. The synthesis of mRNA occurs through the transcription of a DNA template that has been synthesized after the global dissemination of the genetic sequence encoding the immunogen [20]. Once the antigenic sequence is identified, it will need just a few weeks to create and manufacture mRNA-based vaccines on a clinical level. The process of producing mRNA is cell-free and involves in vitro transcription (IVT) [21]. Substantially available materials may be utilized in the laboratory to generate both the template and transcript. Furthermore, a facility designed explicitly for mRNA production ought to possess the capability to rapidly produce vaccines targeting numerous targets with minimal adjustment required [22].

From a business perspective, the very efficient transcription reaction in vitro of the mRNA vaccine makes it possible for large-scale manufacturing and quick development using a cell-free method. Significantly, while mRNA vaccines still have several drawbacks in comparison to other vaccine modalities (e.g., potent immunogenicity and poor stability, which limit their use in vivo), modifications and delivery advancements have substantially mitigated these challenges, ensuring in vivo stability and a balance between inducing robust immune responses and irreversible adverse reactions resulting from prolonged function [23]. mRNA vaccines targeting COVID-19 represent the initial biological preparations authorized for development on this platform. Their deployment has shown to be a crucial intervention that saves lives throughout the epidemic. Moreover, researchers have reported significant progress in clinical trials of mRNA vaccines targeting various viruses, including influenza, HIV-1, respiratory syncytial virus, Nipah virus, Zika virus, human cytomegalovirus, and Epstein-Barr virus. These advancements have occurred since the initial development of the mRNA platform [24-27]. To date, the most advanced products in clinical use consist of non-replicating mRNA vaccines that incorporate both chemically modified and unmodified nucleotide bases. mRNA-1273 from Moderna and BNT162b2 from Pfizer–BioNTech are both approved mRNA products that encompass vaccines composed of chemically modified uradine bases[28].

A pathogen infection (HPV) can be prevented and treated alternatively with a multi-epitope vaccine (MEV), which has been developed continuously in the form of recombinant subunit protein or mRNA vaccines [30]. Platforms comprised of nucleic acid vaccines are effortless to implement, fast to protect and capable of eliciting effective adaptive responses. Nevertheless, it is frequently imperative to employ methods that enhance the immunogenicity of DNA vaccines by preventing the degradation of messenger RNA (mRNA) molecules and facilitating their uptake by immune cells. By utilizing delivery systems that enable the assimilation of particular antigens and assist in modulating the immune response, this limitation may be circumvented [31].

In conclusion, there has been limited research on nucleic acid vaccines in the context of HPV, and their efficacy in animal experiments has been inconsistent. At this time, mRNA vaccines are gaining traction in the context of the epidemic due to their high level of safety and effectiveness [32]. Thus, the various attributes, preventive strategies, and custom-made mRNA vaccines for HPV shall be investigated in this research endeavor.

**2. Characteristics of HPV**

Papillomaviruses are compact, non-enveloped, icosahedral viruses with a diameter of 55 nm. They possess a double-stranded, circular DNA genome that is around 8000 base pairs in length. [33]. These tumors may be seen in a wide range of higher animals, including mammals and birds, affecting the skin and mucous membranes [34]. The tumors they induce vary in kind, ranging from non-cancerous growths such as warts, papillomas, and condylomas, to cancerous growths, primarily squamous cell carcinomas [35]. Due to the severe infections and illnesses they cause in the anogenital tract, a subset of approximately 40 of these viruses has become very important in public and medical health [2].

CC is caused by a persistent infection with one or more genotypes of oncogenic HPV, which is found in the majority of patients with cervical carcinoma [36, 37]. A comprehensive assemblage of over 200 distinct HPV genotypes has been identified and categorized according to their propensity to induce cancer as high-risk HPV (HR-HPV) or low-risk HPV (LR-HPV). Particularly prevalent in invasive CC on a global scale were the HPV genotypes 16, 18, 31, 33, 35, 45, 52, and 58. In the interim, it was documented that there were substantial variations in the distribution of HPV genotypes across countries, including within regions of the same nation [38].

Sexually transmitted diseases can be transmitted through direct sexual contact, including vaginal, anal, and oral intercourse, from over 40 different varieties of HPV [40, 41]. HPVs cause a variety of malignancies by replicating in stratified squamous epithelia. The present scientific endeavors in the field of HPV biology are centered on comprehending the dynamics between the virus and the host, which facilitate HPV's ability to endure in the tissue for years or even decades [42].

HPVs are classified into five genera based on changes in the sequence of the L1 open reading frame (ORF), which contains the genetic information for the main capsid protein. The types of papillomaviruses may be categorized into several groups: Alpha-HPV, Beta-HPV, Gamma-HPV, Mupapillomavirus (mu-HPV), and Nupapillomavirus (nu-HPV). While most of these viruses belong to the Gamma-HPV, Alpha-HPV, and Beta-HPV groups [44]. A further distinction made historically between HPVs has been made regarding their affinity for mucosal or cutaneous epithelia. Therefore, in the literature, Beta- and Gamma-HPVs are characterized as "cutaneous," whereas Alpha-HPVs are still referred to as "mucosal." Nonetheless, mounting data indicates that "cutaneous" HPV is widely distributed at mucosal locations [45-47]. Alpha-HPVs are categorized into two groups: HR and LR, according to the International Agency for Research on Cancer (IARC) carcinogenic potential [48]. The HR types of HPV are unique in their oncogenic capacity due to their role as causative agents of head and neck squamous cell carcinoma (HNSCC), vulvar, penile, and vulvar cancer. More specifically, the development of oropharyngeal squamous cell carcinoma (OPSCC) is mainly attributable to HPV [49]. The percentage of cases in which HPV is responsible for the neoplasms mentioned above varies from 25% (vulvar carcinoma) to almost 100% (cervical and anal carcinomas). In particular, HPV-16 is the most prevalent form among all malignancies associated with HPV, accounting for 60–90% of cases in conjunction with HPV-18 [21]. Beta-HPVs are found in the hair follicles of the genital epidermis, forehead, back of the hand, buttocks, and buttocks, which constitute their natural reservoir. However, as previously stated, the dissemination of Beta and Gamma HPVs also encompasses anatomical sites outside the skin, including the nasal mucosa, oral cavity, and anal canal [50].

The genomes of HPVs are circular, about 7.9 kb double-stranded DNA, with eight major expressed protein-coding ORFs, an intergenic noncoding region (NCR) containing simple (AT)_n_ and poly-T repeats, and an upstream regulatory region (URR). The ORFs are designated E6, E7, E1, E2, E4, E5, L2, and L1 (listed 5′–3′) based on the estimated time of their expression throughout the viral life cycle, where "E" indicates early and "L" indicates late. At certain phases of infection, E8—a sequence often 12 ⅔ codons in length—is spliced to E2 to generate E8^E2, in addition to the significant ORFs. All ORFs are expressed as polycistronic (multi-ORF) mRNAs and are located on the sense (forward) strand [51, 52]. Two polyadenylations (pA) signals, viral early (pAE) and viral late (pAL), can be used to divide the genomes of all papillomaviruses into three distinct regions: an URR, an early (E), and a late (L) gene region. Since HPV does not produce its RNA polymerase, it relies on the transcription of the host RNA polymerase II. Therefore, the functionality of viral promoters is entirely governed by chromatin modifications and transcription factors of the host and virus. Using host polyadenylation machinery, the viral transcripts originating from each promoter are subsequently polyadenylated, either at an early pAE or a late pAL site. pAE and HPV early promoters become active during the early phase of the viral life cycle, while pAL and viral late promoters become active during the late phase [52, 53]. More precisely, the incorporation of E6/E7 genes from the HPV genome into the genetic material of the host, followed by the expression of these genes by the host, maintains an altered phenotype characteristic that has been linked to the formation of cancer [54]. HPV can attack mucous membranes and change the cells in the cervix. This is caused by a sexually transmitted disease [55]. The oncogenes E6 and E7 are essential to HPV infection. Finding these genes to identify HPV strains, especially the HPV-16 strain, would have a substantial effect because of its outstanding sensitivity ; the dielectric electrochemical biosensor stands out among other pathogen detection techniques [56]. New data shows that E6 and E7 are also crucial for stopping the host cell's natural defensive response to HPV [57]. Together with other biological cues, the E1 and E2 proteins drive viral propagation. Additionally, E2 has been connected to the control of transcription in cells and viruses. The purpose of other viral proteins is still unknown after decades of study [58] (Figure 1).

**
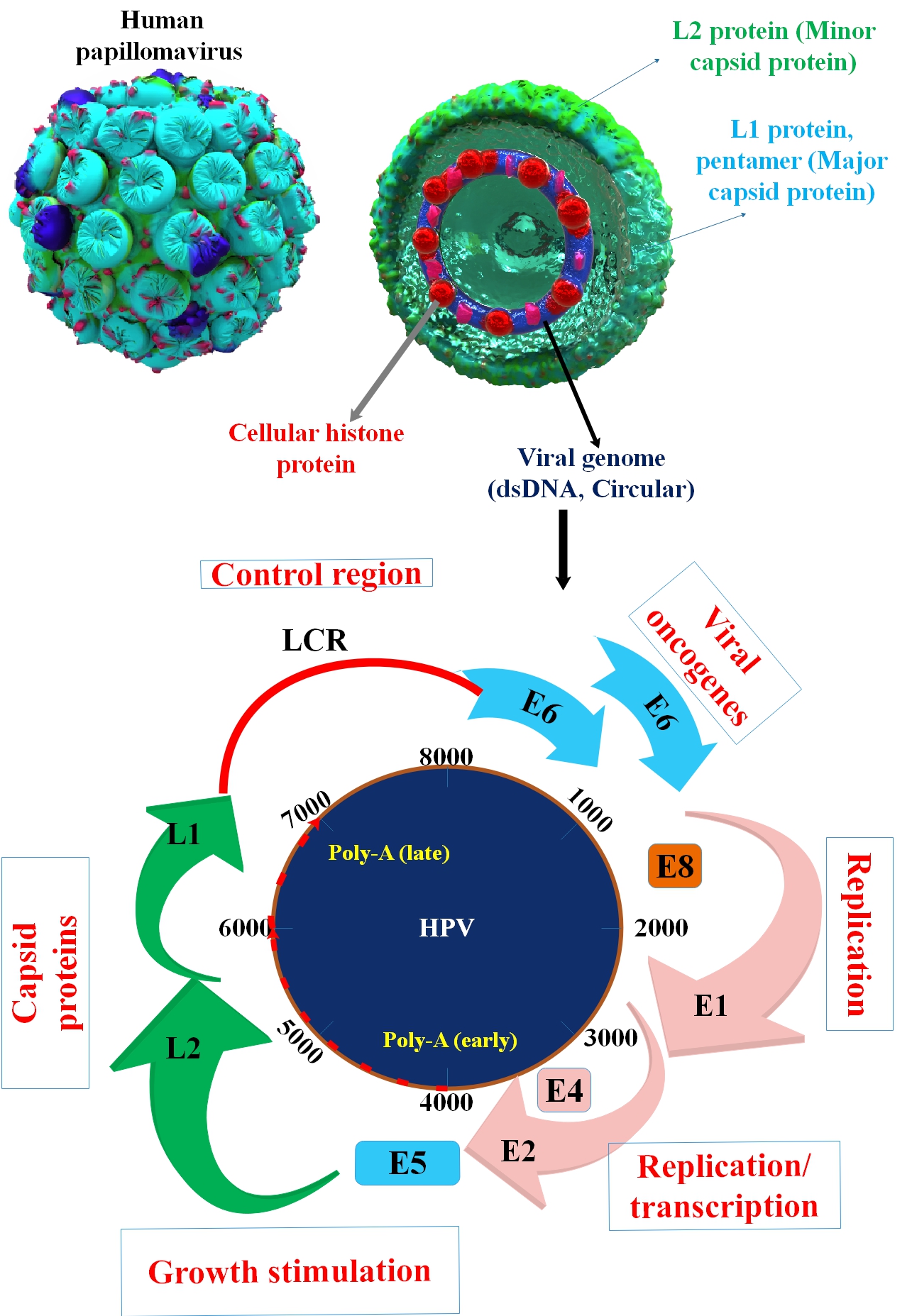
**

**Figure 1.** The genetic makeup and organization of HPV. An illustration of the genetic map of HPV-16 is shown. Open reading frames (ORFs) are denoted by solid bars. The six initial ORFs (E1, E2, E4, E5, E6, and E7) are expressed at distinct phases of epithelial development. The L1 and L2 ORFs are actively transcribed in cells undergoing viral DNA replication in highly differentiated epithelial cells [59]. Three sense-strand trinucleotide (codon) reading frames represent the approximately 7.9 kb circular double-stranded DNA genome of HPV16. Rectangles of color in the appropriate reading frame represent protein-coding ORFs. The three primary parts of the HPV genome are the long control region (LCR), the early and late regions, and the timing of viral protein production after viral entrance into the host cell. The early section primarily encodes regulatory proteins that are necessary for the transcription and replication of the virus as well as cell cycle regulation, which supports HPV's ability to transform and become immortal. The two viral structural proteins, L1 and L2, required for capsid formation, are encoded in the late region. The majority of the regulatory DNA sequences, including the origin of DNA replication and enhancer and promoter regions, that are required for both viral gene expression and genome replication are found in the LCR [60]. E8 is completely encoded within E1, while E4 is completely encoded within E2. The E4 protein is classified as a regulatory protein, which is produced by the genes of early viruses. E1, E2, E4, E5, E6, and E7 proteins are responsible for viral genome replication and maintenance in infected cells. The designations for early and late promoters (p) are p97 and p670, whereas the designations for early and late polyadenylation sites (polyA) are polyAE and polyAL, respectively [51, 61].

**2.1. HPV vaccination methods**

HPV vaccines are some of the best vaccines out there. They were the first to stop infection by a sexually transmitted virus that affects the mucosa without specifically boosting mucosal immunity [95]. Currently, the Chinese market offers five different HPV vaccine types for private purchase: Cervarix® (imported HPV-2), Gardasil® (HPV-4), Gardasil®9 (HPV-9), Cecolin® (domestic HPV-2), and a recently developed bivalent vaccine made domestically called Waldrinvax^TM^, Recombinant HPV Bivalent (Types 16, 18) [96].

In 2006, the first HPV vaccine got its stamp of approval. All vaccines are made to protect against HPV-16 and HPV-18, which are the kinds that cause most cancers linked to HPV. It was 2020, and HPV shots were now part of government vaccination programs in over 100 countries [97]. HPV infection may be prevented by vaccination, and therapeutic vaccination offers the chance to develop cellular immunity against pre-existing HPV infections and lesions as well as stop the spread of malignancy [98]. The HPV vaccines currently on the market are based on virus-like particles (VLPs) that naturally come together from the L1 major capsid protein [99]. The HPV vaccines currently on the market (Gardasil, Gardasil 9, and Cervarix) are meant to protect against HPV and are made from L1-based VLPs that have been added to other substances [100]. The nonavalent vaccination Gardasil 9® was approved in 2014 and targeted the following HPV strains: HPV-6, HPV-11, HPV-16, HPV-18, HPV-31, HPV-33, HPV-45, HPV-52, and HPV-58 [101, 102]. These three well-established vaccines use eukaryotic producer cells [103].

Nonetheless, there is still a market for HPV vaccine development, and Cervavac, a quadrivalent vaccination, was just introduced in India [104]. Based on VLPs, a recombinant, noninfectious assembly of the L1 HPV capsid protein, the vaccines work similarly. Because VLPs and infection-causing HPV virions have the same antigens, exposure to VLPs triggers a potent neutralizing antibody response that prevents HPV from being absorbed by the cervix's basal epithelial cells. The vaccine's effectiveness is due to this humoral reaction [105].

A worldwide double-blind experiment with women aged 16 to 25 evaluated the effectiveness of the nonavalent Gardasil® 9 vaccination. More than 14,000 volunteers were recruited, and in contrast to the previous studies, the quadrivalent Gardasil® HPV vaccination was administered to the control group. The effectiveness of the vaccination against high-grade cervical illness linked to HPV-31, HPV-33, HPV-45, HPV-52, and HPV-58 (i.e., HPV subtypes not protected by the quadrivalent vaccine) was 97.1%; the nonavalent group had 0.5 cases per 10,000 person-years, while the quadrivalent group had 18.1 instances. The two participant groups had similar abnormalities related to HPV-6, HPV-11, HPV-16, and HPV-18 (i.e., HPV subtypes covered by the quadrivalent vaccination). Additionally, the nonavalent vaccination exhibited antibody response for up to five years, and its immunogenicity against HPV-6, HPV-11, HPV-16, and HPV-18 was similar to that of the quadrivalent vaccine. The nonavalent vaccination, according to the scientists, may be able to prevent more incidences of CC by offering more extensive coverage and sustained high effectiveness against all vaccine HPV subtypes [105-107].

The VLPs cause large titers of virion-neutralizing antibodies and resemble real viruses visually. GSK and Merck & Co. were the two firms that started the first commercial development of HPV vaccinations. GSK created Cervarix, a bivalent vaccine comprising HPV-16 and HPV-18 VLPs. Merck created the first quadrivalent vaccination, Gardasil®, and it was approved in 2006. It targets HPV-6, HPV-11, HPV-16, and HPV-18 and protects against genital warts (GW), which HPV-6 and HPV-11 mostly cause [108].

The adjuvants and the producer cells for the viral L1 proteins are further distinctions between the two vaccinations. The proteins for Cervarix and Gardasil are made in yeast (*Saccharomyces* *cerevisiae*) and L1-recombinant baculovirus-infected insect cells, respectively [109]. Cervarix®, a bivalent vaccination that targets HPV-16 and HPV-18, was approved in 2007. While Gardasil includes an aluminum salt adjuvant (aluminum hydroxyphosphate sulfate), Cervarix features a patented adjuvant called AS04, which is made up of aluminum hydroxide with 3-deacylated mono-phosphoryl lipid A, a detoxified form of lipopolysaccharide, and a Toll-like receptor four agonists. Later, Merck created Gardasil 9, a nonavalent vaccination that is comparable to Gardasil but contains L1 VLPs of five additional carcinogenic types: HPV-31, 33, 45, 52, and 58. As a result, Gardasil 9 may provide type-specific protection against 90% of CC cases globally. More recently, producers in China, India, and other nations have begun developing L1 VLP HPV vaccines [110]. The WHO is now reviewing Cecolin, a bivalent vaccine produced by Xiamen Innovax Biotech and contains both HPV-16 and HPV-18 VLPs. The vaccine was approved in China in 2020. *Escherichia coli* (*E. coli*) produces the L1 proteins, and the vaccine contains an alum adjuvant [111].

In many Chinese locations, the bivalent Cecolin® vaccination effectiveness study included more than 7,000 female volunteers aged 18 and 45 in 2012 and 2013. The hepatitis E vaccination was given to the control group. 100% and 97.3%, respectively, of the vaccines were effective against high-grade genital illness and chronic infection linked to HPV-16 or HPV-18. To enable a subgroup analysis, the participants were age-stratified into two groups: 27–45 and 18–26. The Cecolin® vaccine is undergoing a phase 3 clinical study in Ghana and Bangladesh; findings are anticipated in 2023 (NCT04508309) [105, 112].

These and five other HR-HPV strains (31/33/45/52/58), which together account for around 90% of cervical and other HPV-related malignancies, are protected against by the nine-valent HPV (9vHPV) vaccination. Ninety percent of instances of HPV-related GW are caused by HPV-6 and 11, which are also protected against by the 4vHPV and 9vHPV vaccinations. To prevent specific HPV-related illnesses and malignancies, HPV vaccines were initially approved in 2006 (4vHPV), 2007 (2vHPV), and 2014 (9vHPV) after their effectiveness, immunogenicity, and safety were established in clinical studies. Adults and adolescents who are older are also being considered for catch-up vaccinations. In the European Union, the 9vHPV vaccination is recommended for persons nine years and older, with no maximum age restriction. It protects against precancerous lesions and malignancies of the cervix, vulva, vagina, and anus caused by particular forms of HPV, as well as GW produced by a specific HPV type. The 9vHPV vaccine is authorized for use in females aged 9-45 in the US to prevent cervical, vulvar, vaginal, and anal (pre)cancers, as well as oropharyngeal and other head and neck cancers and GW. It is also approved for males aged 9-45 to prevent anal (pre)cancer, oropharyngeal and other head and neck cancers, and GW. Adults may benefit from HPV vaccinations as well, even though young teenagers are the main target population for immunization. Regardless of an adult's HPV infection status, the US Advisory Committee on Immunization Practices advises regular catch-up immunization through age 26 and shared clinical decision-making about HPV vaccination until age 45 [113].

The countrywide implementation of HPV vaccination often encounters challenges, such as poor compliance with administering the second or third dosage and insufficient funding to provide the total doses of the HPV vaccine, particularly in low- and middle-income countries. Administering a single dose of an HPV vaccine has the potential to address these concerns and substantially mitigate these challenges effectively. Before making a choice, it is crucial to have reliable and measurable information about the effectiveness of a single-dose HPV vaccine. A systematic review compares the efficacy of a single dose of the HPV vaccine (one dose) to that of two and three doses regarding infection prevention and pre-cancer incidence. The majority of the studies included in this systematic review and meta-analysis provide evidence that a single dose of an HPV vaccine can generate HPV-specific antibodies for a duration of up to 8 years. This establishes immunogenic memory and demonstrates that a single dose is equally effective in preventing infection and pre-cancerous conditions as receiving two or more vaccine doses. Nevertheless, there is limited research elucidating why antibodies do not respond similarly to two or three dosages, and neither the incidence of pre-cancer nor the infection rate is significantly reduced. Consequently, further research and longer durations of study are necessary to establish unequivocally the efficacy of the one-dose HPV vaccine [96] (Table 1).

**Table 1.** HPV vaccines are currently licensed in the United States.

| **Vaccine** | **Recommendation of 1 or 2 doses** | **Three doses** | **Year** | **Manufacturer** | **HPV type** | **Ref** |
| --- | --- | --- | --- | --- | --- | --- |
| Bivalent 2v HPV (Cervarix) | Two doses spaced six months apart may be administered to individuals 9 to 14. The second dosage may be administered between 5 and 13 months after the first dose if required. | Individuals 15 and older are administered three doses.series:0,1,6 month | October2009, females | GlaxoSmithKline | 16,18 | [114-116] |
| Quadrivalent 4v HPV (Gardasil) | a two-dose course (0, 6–12 months) for most people who start vaccinations between the ages of 9 and 14. | A three-dose series (0, 1-2, 6 months) is recommended for individuals aged 15 to 45 who begin vaccinations, as well as for those who are immunocompromised. | June 2006 females, October 2009 males | Merck | 6,11,16,18 | [117-119] |
| 9-valent9v HPV(Gardasil9) | As a single-dose vial or prefilled syringe (0.5 mL), HPV-9 is offered for purchase. Individuals under the age of 25 typically receive one dose. Individuals aged 25 to 45 usually receive two injections, spaced between two years and six months. | Individuals with compromised immune systems get three treatments over an entire year. | December 2014, females and males | Merck | 6,11,16,18,31,33,45  52,58 | [117, 120-122] |

**3. mRNA** **vaccine engineering**

The differences in immunological responses to these various vaccines have been the subject of numerous studies, particularly comparing the reactions to the bivalent and quadrivalent vaccines. Considerable research has been devoted to examining antibody responses, given their critical role in mediating protection against infections. Although antibody concentrations diminish and memory immunity is formed and maintained through the presence of specific memory B and T cells, cell-mediated immunity to HPV antigens remains a crucial component in the fight against HPV infection. In addition to innate cells that participate in antigen presentation and early defense, cellular immunity also encompasses adaptive responses in which T helper cells facilitate the production and (re)activation of B cells, thus facilitating the production of high levels of antibodies. It has been demonstrated that all three vaccines elicit robust antibody responses against the different vaccine types; however, the bivalent vaccine elicited more significant quantities of HPV16/18-specific serum antibodies and more robust B-cell responses. This was primarily attributed to the AS04 adjuvant, which is believed to stimulate a more favorable Th1 response [123]. Initial and subsequent investigations evaluating the immunogenicity of the HPV 16/18 AS04-adjuvant vaccine in women aged 15 to 25 revealed that total IgG antibodies against HPV16 and anti-HPV18 peaked at month 7, reached a plateau between months 18 and 24 and remained stable for up to 76 months following vaccination. High concentrations of functional antibodies were further validated by using pseudovirion-based neutralization assays to measure the neutralizing antibodies. Then, 10 years after the initial vaccination, an assessment of the long-term immunogenicity of the HPV16/18 vaccine in the serum of females aged 15 to 55 revealed that anti-HPV16 seropositivity remained elevated in all age groups. Seropositive females for anti-HPV18 were more prevalent among those aged 15 to 25 (99.2%) compared to those aged 26 to 45 (93.7%) and 46 to 55 (83.8%) [124, 125]. Although the three approved vaccines exhibited comparable effectiveness against HPV infection and precancerous lesions in clinical trials, they diverged in immunogenicity, as confirmed by an array of assays. In head-to-head trials comparing three doses of Cervarix® and Gardasil® in females aged 18–45 and females aged 12–15, HPV-16 antibody levels were substantially lower in females aged 12–15 treated with Gardasil® compared to Cervarix®, although their patterns of peak and decline over time were comparable. In comparison to Cervarix®, HPV-18 antibody levels and seropositivity were notably diminished in Gardasil®. Additionally, memory B cell responses were diminished, specifically for HPV-18, and HPV-16 and HPV-18 specific CD4+ T cell responses were reduced by Gardasil® for up to 24 months following vaccination [126-128]. The goal of HPV prophylactic vaccinations is to prevent HPV infection by stimulating the body's humoral immunity to produce neutralizing antibodies. Nevertheless, an organism that has already acquired the virus cannot be effectively treated with the HPV prophylactic vaccination. Furthermore, several early genes (E1, E2, E4, E5) and late genes (L1, L2) have been lost as a result of the integration of the viral genome into the host genome, rendering preventative vaccinations useless against HPV-related precancerous lesions and malignancies [32].

As of right now, the most extensively used vaccination is the mRNA vaccine, which has shown to be an auspicious treatment approach in immunotherapy. Malone et al. showed in 1989 that encapsulating a cationic lipid (N-(1-(2,3-dioleyloxy)propyl)-N, N, N-trimethylammonium chloride (DOTMA)) could efficiently transfect and express mRNA in a variety of eukaryotic cells [129]. Following this, complete expression of mRNA transcribed in vitro was observed in mouse skeletal muscle cells in 1990. This event marked the initial instance of practical in vitro mRNA expression, which validated the viability of mRNA vaccine development [130]. Prioritizing the development of DNA-based methods was necessary because of RNA's instability, ineffective in vivo delivery, and tendency to trigger exaggerated inflammatory responses until the late 2000s. Vaccines targeting mRNA are a relatively new vaccine type that has a lot of potential [131]. A coding sequence, a 3' poly A tail, a 5'cap structure, and 5' and 3' untranslated regions (UTR) structures make up the mRNA structure [18].

Numerous studies have demonstrated that mRNA is unintegrated (safe) and that the capacity for autonomous replication is exceptionally high in the new generation of self-amplifying mRNA vaccines (saRNA vaccines). The significant factors contributing to the comparatively sluggish advancement of mRNA vaccines are their inadequate stability and delivery efficiency in contrast to DNA vaccines. As a result, delivery vectors, such as DC vectors, protamine, cationic lipid delivery systems, and polymer materials, are frequently used to transport mRNA into the body [132]. mRNA technology is a desirable substitute for conventional or even DNA vaccines since it offers several benefits. mRNA is precise because it will only express a particular antigen and trigger a tailored immune response, unlike attenuated or inactivated vaccines [133].

Additionally, it induces the innate immune system and stimulates humoral and cellular immune responses. mRNA vaccines are safer and more effective than DNA-based vaccines since expression does not necessitate nuclear entry; the likelihood of random genome integration is virtually non-existent [18, 134]. Additionally, since mRNA is rapidly broken down by cellular processes and disappears within two to three days, expression of the coded antigens is only temporary [135]. The manufacturing process benefits from the adaptable nature of the mRNA vaccine platform as well; modifications to the encoded antigen do not impact the physical-chemical properties of the mRNA backbone, enabling standardized production. Furthermore, the utilization of an in vitro cell-free transcription reaction for production mitigates safety apprehensions associated with the potential presence of viral contaminants and cell-derived impurities that are frequently encountered on alternative platforms [136].

Because the mRNA is prone to degradation, stabilizing it might increase expression. The expression and stability of mRNA vaccines are influenced by several variables. The stability and translation efficiency of mRNA is directly influenced by its structural features, including the CAP, poly(A) tail, and UTRs. For mRNA to remain stable in the cytosol, the poly(A) tail and cap, found at the 5′ and 3′ ends of mRNA, are essential [140]. To circumvent this, mRNA can be delivered via dendritic cell transfection, conjugation with polymers, peptides, or lipid-based carriers, injection of bare mRNA, or conjugating mRNA with lipid-based carriers [142]. The four material types—polymers, peptides, protamines, and lipids—as well as their corresponding delivery methods will be covered by the mRNA vaccine delivery systems. This will improve the stability, effectiveness, and safety of the naked mRNA once it is administered [143-145].

Moreover, therapies and vaccines based on mRNA are produced via rapid, cell-free IVT. Synthetic mRNA is generated by transcribed in vitro by an RNA polymerase from a plasmid DNA (pDNA) template that has been prepared and linearized. The resulting mRNA typically consists of a 5′ cap and a template-encoded 3’ poly(A) tail. Additionally, antisense and double-stranded RNAs, which can trigger an innate immune response that inhibits cellular translation and causes adverse effects, must be eliminated from the mRNA [151]. Endosomal or cytosolic receptors within the cells recognize RNA, which may activate the type I interferon (IFN-I) pathway and promote the production of proinflammatory cytokines and chemokines. These signaling molecules cause the activation of antigen-presenting cells (APCs), which in turn triggers a potent adaptive response [150].

mRNA vaccines, instead of peptides, do not impose restrictions on MHC haplotypes. Moreover, since mRNA bonds to pattern recognition receptors, mRNA vaccines have the potential to function as self-adjuvants, a characteristic that is absent in vaccines composed of peptides and proteins. mRNA vaccines combine the untapped versatility of genetic vaccines with exceptional safety and desirable immunological properties [155]. mRNA vaccines are not limited by MHC haplotype and may elicit a balanced immune response that includes humoral and cellular immunity based on in situ protein production. Furthermore, since mRNA is a minimum and only transitory information carrier that does not interact with the genome, it is an essentially safe vector. mRNA vaccines also provide the more significant degree of development flexibility possible, as any protein may be created from mRNA without modifying the manufacturing procedure. When combined, mRNA offers a promising vector that might serve as the cornerstone of a revolutionary vaccine technology platform [156] (Figure 2).


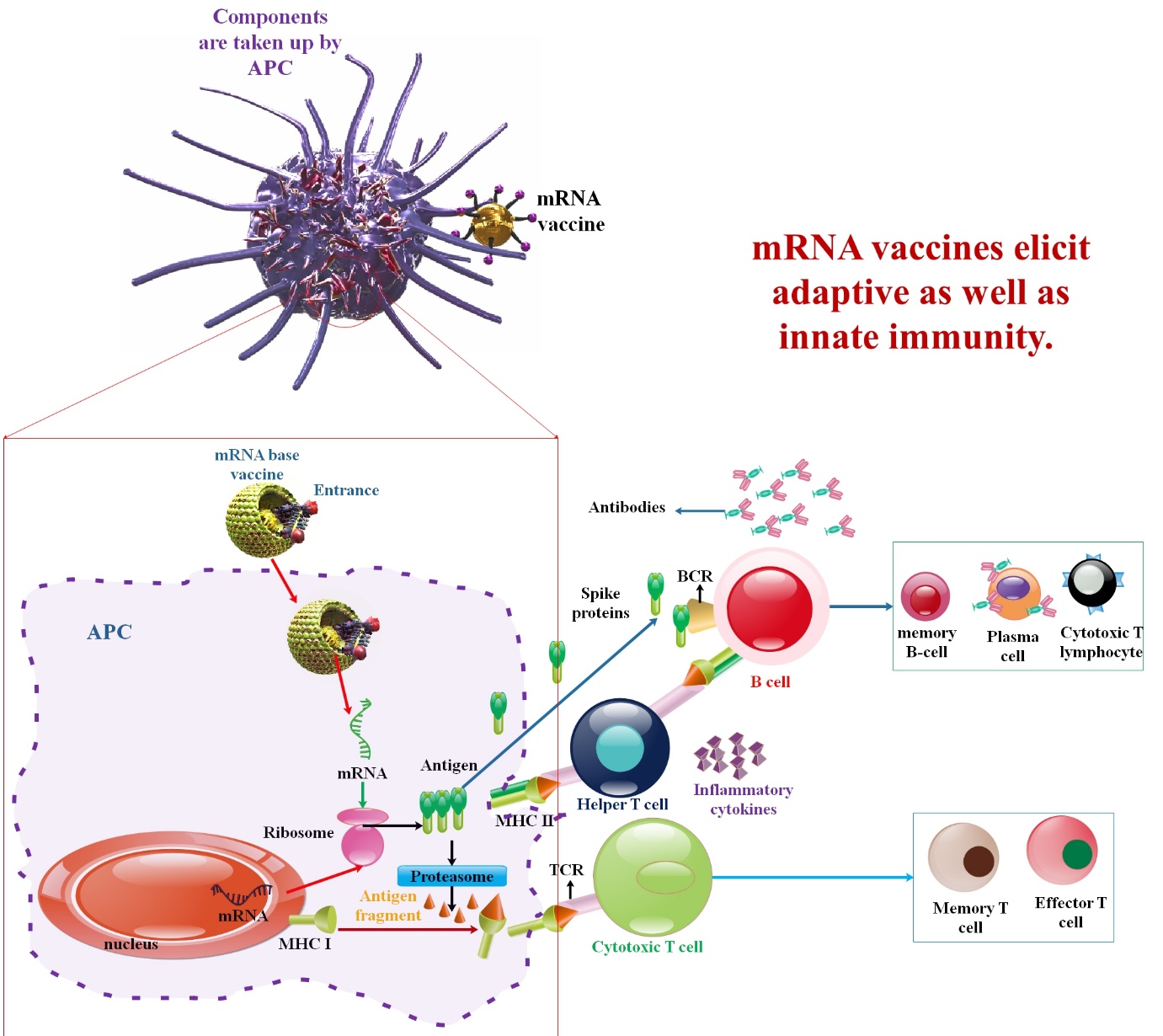


**Figure 2.** mRNA vaccines have dual impacts on immune activation. mRNA vaccines elicit adaptive as well as innate immunity. Antigen-presenting cells detect endocytosis of exogenous mRNA by TLR3 and TLR7/8 in the endosomes and RIG-1, NOD2, LGP2, and MDA-5 in the cytosol. This triggers robust IFN-I responses, which stimulate the production of proinflammatory cytokines and ultimately activate innate immunity (left). To activate CD4+ and CD8+ T cells, which aid in triggering adaptive immunity, peptides transcribed by re-endocytosed mRNA are displayed on MHC-I or MHC-II molecules (right). The release of proteins encoded by mRNA activates B cells [23, 165].

**4. mRNA vaccines in HPV**

mRNA-based therapeutics have enormous potential as clinical remedies; nevertheless, developing methods for delivering the bioactive agents in a safe, efficient, and immune-suppressant manner will be a significant obstacle to the realization of this class of medications [166, 167]. In the context of mRNA vaccines, scientists have made structural modifications to mRNA to improve its stability and facilitate systemic tolerance to antigenic presentation outside of inflammatory environments [168]. One significant challenge to treating HPV infections in the lower genital tract is the development of therapeutic vaccines. The development of therapeutic vaccines certainly seems like a good idea for medical care. Nevertheless, there aren't any of these vaccines available for clinical use right now [169]. Therapeutic vaccines, in contrast to preventive vaccines, are designed to stimulate an immune response mediated by specific T cells. These devices are purposefully engineered to eradicate any remaining tumor lesions that may remain after an HPV infection, such as condyloma acuminata, a type of cervical atypical hyperplasia [167, 170]. Cervical intraepithelial lesions fall into two categories, as per the 5th edition of the WHO Classification of Female Genital Tumors: high-grade squamous intraepithelial lesions (HSIL), which includes cervical intraepithelial neoplasia grade 2 (CIN2) and CIN3 and low-grade squamous intraepithelial lesions (LSIL), which provides for CIN1. According to research data, after a 10-year follow-up period, 20–30% of HSIL cases may develop into invasive CC [171-173]. Currently, available treatments for LISL include lesions being removed and monitoring. With a 60% natural regression rate, LSIL has a high lesion persistence rate of 30% and a 10% progression rate to HSIL. Another meta-analysis revealed that 21% of LSIL will proceed to HSIL, 0.15% of LSIL will progress to an invasive cervical malignancy, and 48% of LSIL cleared spontaneously [174, 175]. Live vector vaccines are composed of viral or bacterial vectors. *Listeria monocytogenes*, *Lactobacillus casei*, and *Lactococcus lactis* are frequently utilized bacterial vector-based vaccines. The recombinant L. casei vaccine GLBL101c, which expresses HPV-16 E7, showed an E7-specific immune response and downgraded CIN3 lesion in a phase IIB study. The effectiveness of the alphavirus, adenovirus, and bovine pox virus has also been studied in clinical studies. However, the possible pathogenicity of the vectors and the existence of neutralizing antibodies, which restricts recurrent treatment, provide difficulties for using live vector vaccines in immunocompromised individuals [167, 170].

As determined by researchers, persistent HPV infection is a leading cause of cancer-related mortality in women, and there is an urgent need for the development of safe and effective therapeutic strategies. A comparison was made between three distinct mRNA vaccine modalities to assess their efficacy in targeting tumors that are linked to HPV-16 infection in mice. Self-amplifying mRNA encapsulated in lipid nanoparticles (LNPs), unmodified and nucleoside-modified non-replicating mRNA vaccines containing a chimeric protein formed by the fusion of the herpes simplex virus type 1 glycoprotein D (gDE7) and the HPV-16 E7 oncoprotein, were all produced by the researchers. It was shown that a single low-dose vaccination with any of the three gDE7 mRNA vaccines activated CD8+ T cells specific to E7, produced memory T cell responses that could stop tumor relapses, and eliminated subcutaneous tumors at various stages of development. Furthermore, after the administration of a single vaccination dosage, the gDE7 mRNA-LNP vaccines produced strong tumor protection in two distinct orthotopic mouse tumor models. Finally, comparison experiments showed that gDE7 mRNA-LNP vaccines, all three of them, outperformed gDE7 DNA and gDE7 recombinant protein vaccines [176].

Further examination reveals that while mRNA vaccines are acknowledged for their capacity to provoke immune responses targeted against infectious diseases, their impact on the functional commitment of CD8+ T cells in the tumor microenvironment (TME) and secondary lymphoid organs is still not well comprehended. This lack of understanding limits their potential for broader application in cancer immunotherapy. An investigation aimed to thoroughly assess the immune effects of an HPV E7 protein (HPV mRNA-LNP), a tumor-specific antigen of HPV-positive OPSCC, encoded in an LNP-encapsulated mRNA vaccine. HPV mRNA-LNP vaccination induced different patterns of immune response and fatigue in the spleen and TME. This vaccination had varying effects on the functional specialization of CD8+ T cells and activated both HPV-specific and total CD8+ T cells. The concurrent administration of immune checkpoint blockades and HPV mRNA-LNP vaccination increased HPV-specific CD8+ T cells while preserving their antitumor capabilities, thereby facilitating additional tumor regression. The combination of HPV mRNA-LNP vaccination and immune checkpoint blockade is a promising immunotherapeutic strategy for HPV-positive OPSCC, according to the findings of the researchers [177].

As they modulate cell cycle regulation, the E6 and E7 proteins of particular subtypes of HPV, including HPV-16 and 18, are strongly associated with CC, according to another study. The objective of this research endeavor was to examine the potential antitumor properties of a therapeutic vaccine (mHTV) composed of mRNA-HPV and non-oncogenic E6 and E7 proteins. To accomplish this, the vaccine was administered subcutaneously and intramuscularly to C57BL/6j mice, and the subsequent effects were assessed. In both subcutaneous and orthotopic tumor-implanted mice models, mHTV vaccination dramatically enhanced T cell-mediated immune responses and considerably inhibited tumor development, with a notable infiltration of immune cells into tumor tissues. In all mHTV-treated animals, tumor retransplantation at day 62 postprimary immunization stopped the tumor's development. In addition, TC-1 transplantation substantially inhibited tumor growth 160 days after the most recent vaccination. mHTV-induced immunization of rhesus primates produced encouraging immune responses. The immunogenicity of mHTV in nonhuman primates provides solid support for its potential clinical application in humans to combat malignancies associated with HPV. Based on all available data, mHTV exhibits potential as a prophylactic and therapeutic vaccine [178].

For several reasons, the carcinogenic early proteins E6 and E7 are the most often targeted antigens by the HPV16 therapeutic vaccines now under development. 1) Since precancerous and cancerous lesions are the only places where E6 and E7 are expressed in large quantities, there is no significant risk that they will target healthy tissues; 2) Since E6 and E7 are necessary for the transformation and maintenance of infected cells, there is no significant risk that antigen loss-mediated immune escape will occur; 4) The immune response to E6 and E7 has been described preclinically and clinically; 3) no mechanisms of central tolerance against E6 and E7 have been revealed. Currently, the US FDA has not authorized any therapeutic HPV vaccination. Here, scientists present an mRNA-based vaccination that encodes for altered forms of the HPV16 early proteins E6 and E7. The vaccine is prepared as a lipid nanoparticle (LNP) based on MC3, and it may be injected intramuscularly. According to in vivo and in vitro research, the translated mRNA is functional and elicits a distinct adaptive immune response to the antigen. Vaccinated mice harboring HPV16+ lesions exhibited reduced tumor growth, extended lifespans, and the development of a protective immunological memory. A therapeutic vaccine based on mRNA has the potential to provide a non-invasive alternative to the current standard of care for HPV16+ HSILs, according to researchers. The oncogenic early proteins E6 and E7 are widely recognized targets of HPV16 vaccination strategies. As mentioned, E6 and E7 can be modified with deletions and mutations, respectively, to prevent them from binding to p53 and pRb, thereby eradicating their potential for oncogenesis. A highly efficient IgE leader sequence is encoded in the mRNA of the HPV-16 vaccine developed by researchers. This sequence aids in the expression of a fusion protein that comprises the modified E6 and E7, which are partitioned by a furin cleavage site [179].

According to another research, efforts to fight HPV are still being made via the development of recombinant protein-based vaccinations. The primary capsid protein L1, which may clump together to form VLP and trigger strong immunological reactions, is the protein most often exposed to the immune system. Cervarix and Gardasil, the two primaries approved and commercially available HPV vaccines, now use systems based on pure recombinant VLP. Using *E. coli* as an example, the HPV L1 protein might be produced in prokaryotic, eukaryotic, insect, plant, and yeast systems, among others. Scientists persist in developing MEV primarily due to its cost-effectiveness, high efficacy, and safety. At present, the most efficacious vaccine against HPV is one based on VLPs; these vaccines can be manufactured using a variety of expression systems. The study centers around a comparison of the recombinant protein expression of L1 HPV-52 using two widely used yeast strains, namely *Hansenula polymorpha* and *Pichia pastoris*, both of which have been employed in the industrial production of vaccines. Reverse vaccinology, a bioinformatics technique, was also used by researchers to create alternative MEVs for recombinant protein and mRNA types. Compared to H. polymorpha, P. pastoris exhibited a greater degree of L1 protein expression and production efficiency in a batch system, according to the researchers' findings. Nonetheless, upon protein induction, both hosts demonstrated stable integration and the creation of self-assembly VLPs. Researchers have developed vaccines that are safe to forecast computationally and have significant immune activation. Additionally, it could work well for manufacturing in a range of expression systems. Large-scale manufacture of the HPV-52 vaccine may be based on this study's monitoring of the overall optimization parameter evaluation [30].

SQZ-eAPC-HPV (eAPC) is an autologous, HLA-genotype-agnostic, cell-based therapeutic cancer vaccine for HPV that utilizes manufacturing and clinical expertise gained from the SQZ-PBMC-HPV clinical candidate (NCT04084951), according to another study. Cell Squeeze® technology is used to engineer eAPC from peripheral blood mononuclear cells to deliver concurrently five mRNAs encoding for the full-length HPV16 E6 and E7 proteins, CD86, membrane-bound (mb) IL-2, and mbIL-12 cytokines. In addition to minimizing the known toxicity of interleukin treatment, the eAPCs may promote more robust T cell activation and proliferation by promoting co-localized MHC-I antigen presentation (E6/E7), costimulation (CD86), and cytokine signaling (IL-2/IL-12). The effects of multiplexed mRNA-based creation of a cancer vaccine are investigated using eAPC [180].

An extra study demonstrates that infections with HPV-16 are linked to a diverse range of malignancies. Furthermore, it provides strong evidence that the transformative capability of HPV-16 is heavily reliant on the expression levels of the viral oncoproteins E6 and E7. Therapeutic cancer vaccines that possess the ability to induce lasting and targeted immunity against these HPV-16 antigens exhibit significant potential in attaining permanent disease management. Researchers demonstrate that intravenously administered cancer vaccine HPV-16 E7 RNA-LPX, which is composed of immuno-pharmacologically optimized antigen-encoding mRNA, primes and expands antigen-specific effector and memory CD8+ T cells in mice. Immunized mice harbor HPV-positive TC-1 and C3 tumors that are densely infiltrated with HPV-16-specific T cells and activated immune cells; these cells are oriented in a proinflammatory, cytotoxic, and less immune-suppressive manner. Immunization against E7 RNA-LPX induces lasting and comprehensive remission of advancing malignancies. Highly cytotoxic memory T cells in circulation protect tumor re-exposure. In addition, checkpoint blockade rendered anti-PD-L1 refractory tumors susceptible to E7 RNA-LPX immunization. In summary, the findings of the researchers underscore the potential of HPV-16 RNA-LPX in mitigating malignancies caused by HPV [181].

According to different research, CIN, a precursor to CC, is mainly caused by infection with HR-HPV strains. Despite the great efficacy of preventive HPV vaccinations, a significant number of women remain at risk for CIN because of accessibility issues, a lack of knowledge, or personal preferences. Although the loop electrosurgical excision process is a successful therapy for many CIN2/3 patients, it may have serious side effects and does not entirely remove the underlying HPV infection. Thus, non-surgical therapies for the management of high-grade CIN represent an unmet medical need. Nutcracker Therapeutics Inc. has created a novel therapeutic vaccination medication candidate called mNTX-250, which is based on mRNA. It includes the HPV-16 E6/E7 oncoproteins housed in the human cluster of differentiation (hCD1d) scaffold, immunomodulators encapsulated in LNPs, and engineered human LIGHT (hLIGHT) and human interleukin-12 (hIL-12). This study aimed to compare the antigen-specific immune memory induction, pharmacodynamics, and in vivo efficacy of mNTX-250, a murine surrogate for NTX-250, and NTX-010, which includes noncoding mRNA, murine IL-12, and murine LIGHT, against HPV-16 antigens in C3.43 tumors. Reintroduction of TC-1 tumors into tumor-free mice occurred. mNTX-250 therapy, administered in two doses, not only eradicated established B6 C3.43 tumors transformed with HPV16 but also enhanced overall survival, but also generated a substantial population of T cells that are specific to the HPV16 E7 antigen. The tumor-free mice were reintroduced to 105 TC-1 tumor cells, which are B6 mouse lung epithelial cells that have been transformed by HPV16 E6/E7, after their treatment with mNTX-250 or NTX-010. While 6/11 NTX-010-treated tumor-surviving mice had TC-1 tumor development, all mNTX-250-treated mice with completely eradicated C3.43 tumors demonstrated total rejection of TC-1 tumors. When given NTX-010, mice with TC-1 tumor development also produced fewer T lymphocytes specific to the HPV16 antigen. In the C3.43 tumor model, mNTX-250 showed strong anticancer effectiveness and produced immunological memory particular to the HPV-16 antigen that was enough to shield the host against the recurrence of another HPV-16-driven tumor [182] (Table 2).

**Table 2.** Explanation of the potential of using mRNA vaccines against HPV.

| **mRNA vaccine** | **Type of HPV** | **Explanation and function of mRNA vaccines** | **Ref** |
| --- | --- | --- | --- |
| gDE7 mRNA | HPV-16 | Administration of any of the three gDE7 mRNA vaccines induced a robust activation of CD8+ T cells that specifically targeted E7. Furthermore, these vaccinations elicited memory T cell responses capable of preventing tumor relapses and effectively eradicating subcutaneous tumors at different stages of growth. | [176] |
| HPV mRNA-LNP | HPV-16 | HPV mRNA-LNP vaccination induced different patterns of immune response and fatigue in the spleen and TME, respectively. This vaccination had varying effects on the functional specialization of CD8+ T cells and stimulated both HPV-specific and total CD8+ T cells. | [177] |
| RNA-HPV therapeutic vaccine (mHTV) | - | The administration of mHTV vaccine elicited strong T cell-mediated immune responses. It effectively inhibited tumor development in both subcutaneous and orthotopic mice models with notable infiltration of immune cells into tumor tissues. | [178] |
| HPV16 E6/E7 -based mRNA vaccine | HPV16+ | Researchers suggest that an mRNA-based therapeutic vaccination has the potential to provide a non-invasive alternative to the current standard treatment for HPV-16+ HSILs. The oncoproteins E6 and E7 are often targeted in HPV-16 immunization methods. | [179] |
| VLP-based vaccine | HPV-52 | Efficacy and safety. At present, the most efficacious vaccine against HPV is one based on VLPs; these vaccines can be manufactured using a variety of expression systems. The study centers around a comparison of the recombinant protein expression of L1 HPV-52 using two widely used yeast strains, namely *Hansenula polymorpha* and *Pichia pastoris*, both of which have been employed in the industrial production of vaccines. | [30] |
| SQZ-eAPC-HPV | HPV16+ | The HPV therapeutic cancer vaccine SQZ-eAPC-HPV (eAPC) is made from the patient's cells and doesn't depend on the HLA gene. The clinical and industrial know-how from the SQZ-PBMC-HPV clinical candidate (NCT04084951) is used. eAPCs are made from mononuclear cells in the peripheral blood using Cell Squeeze® technology. They are programmed to release five mRNAs at the same time, which code for CD86, membrane-bound (mb) IL-2 and mbIL-12 cytokines, and full-length HPV16 E6 and E7 proteins. | [180] |
| HPV-16 E7 RNA-LPX | HPV16+ | Researchers have shown that when mice are given the cancer vaccine HPV-16 E7 RNA-LPX by intravenous injection, it stimulates the production and growth of particular CD8+ T cells that are responsible for targeting and destroying antigens. Mice that have been immunized have HPV-positive TC-1 and C3 tumors that have a high concentration of HPV-16-specific T cells and immune cells that are in an active state. | [181] |
| mNTX-250 | HPV16+ | The objective of an investigation was to assess and compare the ability of mNTX-250, a mouse equivalent of NTX-250, and NTX-010, which contains noncoding mRNA, murine IL-12, and murine LIGHT, to induce antigen-specific immunological memory, evaluate their pharmacodynamics, and measure their effectiveness against HPV-16 antigens in C3.43 tumors. | [182] |

**5. Advantages and disadvantages of mRNA vaccines**

In animal models, mRNA vaccine constructs have been shown in several recent studies to confer protection against an extensive array of infectious agents, including *Streptococcus* sp, *Toxoplasma gondii*, Zika virus, rabies, HPV, influenza virus, cytomegalovirus, and Ebola virus [183]. Studies have shown that mRNA-based vaccine formulations may induce strong immune responses in animals, even with a low number of vaccination doses. When given an mRNA–LNP vaccine containing the genetic information for the pre-membrane and envelope (prM-E) glycoproteins of the Zika virus, non-human primates (NHPs) and small animals showed a strong and durable immune response in the form of neutralizing antibodies. This antibody response bestowed sterile immunity against ZIKV infection [184, 185]. In a similar vein, NHPs that were administered an i.m. vaccination using an mRNA vaccine formulated by LNPs and encoding the rabies virus glycoprotein (RABV-G) generated antibody titers that were stable for one year and capable of being boosted [186]. The study found that NHPs who received a single dose of an mRNA-LNP vaccine containing the genetic code for the hemagglutinin glycoprotein of the H1N1pdm09 influenza virus strain produced levels of anti-H1N1-HI antibodies that were at least as high as, if not higher than, the levels considered protective in humans. These antibody levels were also comparable to those produced by a licensed inactivated influenza virus vaccine called fluad [187]. The outcomes of such animal investigations have sparked considerable enthusiasm. Clinical trials are presently underway to evaluate mRNA vaccines against various viral diseases, such as novel coronavirus (SARS-CoV-2), rabies virus, influenza virus, Zika virus, cytomegalovirus, and respiratory syncytial virus. However, apart from the SARS-CoV-2 trial, none of these clinical trials have progressed beyond the initiation phase [22]. As an illustration, a study demonstrated that scientists have created an mRNA-based vaccine targeting the late oncoproteins E6 and E7 of HPV-16. These oncoproteins are expressed exclusively and abundantly in HSILs, a precursor stage to carcinoma in cervical disease. In vitro and in vivo investigations by the researchers established that the translated mRNA induces an antigen-specific adaptive immune response and is functional. Mice harboring HPV-16+ lesions demonstrated tumor growth inhibition, lifespan extension, and the formation of a protective immune memory after vaccination. Given the aforementioned findings and the notable clinical achievements of mRNA vaccines targeting SARS-CoV2, researchers are convinced that their mRNA-based therapeutic vaccine could furnish an alternative non-invasive therapeutic approach to the existing gold standard for HPV-16+ HSILs [179].

The expeditious manufacturing turnover and comparatively low production costs are additional advantages of the mRNA modality. These factors have played a significant role in the triumph of Spikevax® and Comirnaty®, two mRNA vaccines designed to elicit protective immunity against the SARS-CoV-2 Spike protein. Furthermore, the versatility of mRNA synthesis platforms is enhanced by the potential for mRNA to codify for any protein or peptide; consequently, personalized vaccines are no longer an unattainable fantasy. Two of the existing limitations of researchers and other HPV vaccines are the restriction to single genotype-specific antigens (HPV16's E6 and E7) and the absence of demonstrated overlapping efficacy with other HR-HPV genotypes, which could augment and expand the immune response against HPV lesions. The current vaccine, which targets both E6 and E7, is designed to treat patients with HPV16-associated lesions. In the investigations of Zhou et al., it was observed that patient-derived T cells exhibited an IFNγ response when exposed to putative dendritic cells that express the vaccination protein. This strengthens the confidence of this study regarding the potential for investigators' vaccine to be translated. The transformation of the C3.43 TME into a cytotoxic signature was an additional significant finding. By analyzing the immunogenicity of spleen cells from vaccinated mice, it was determined that antigen stimulation activated cytotoxic T lymphocytes but not helper T cells. A negligible antibody-dependent cellular cytotoxicity (ADCC) that contributed to the elimination of C3.43 tumor cells cannot be ruled out based on the humoral response that was observed. As E6 and E7 are expressed intracellularly within the transformed epithelium of the cervix, the reported humoral immune response elicited by our vaccine is unlikely to have any practical implications in the form of ADCC implementation. In summary, the immune memory induced by our vaccine, either independently or in conjunction with pre-existing tumor reactivities against C3.43 tumor antigens, resulted in tumor rejection in all complete responders without requiring an additional dose. This finding implies that the Zhou et al. vaccine effectively established a durable barrier against recurrence or reactivation. However, the lack of tumor inhibition observed in C3.43 tumor-bearing mice vaccinated with E1, E2, and E5 indicates that E6 and E7 are primarily targeted by the vaccine-induced immune response, which is more prevalent and protective than other potential immune responses not associated with vaccination. In summary, researchers’ therapeutic vaccine utilizing mRNA to target the oncogenic proteins E6 and E7 of HPV-16 exhibits potential as a candidate for subsequent clinical trial evaluation. Further investigations in larger animal species will provide valuable insights into the clinical translational potential of this vaccine. If efficacious in human subjects, their vaccine may serve as a safer and less invasive substitute for surgical intervention among reproductive-aged women afflicted with HSIL [188].

A plethora of evidence not only demonstrated that mRNA facilitates enhanced transfection efficiency and prolonged protein expression time but also unveiled the primary benefits that distinguish mRNA from DNA. Among the benefits of mRNA are: (1) For mRNA to be functional, it is not required to access the nucleus. Upon entering the cytoplasm, mRNA commences the process of protein translation. DNA, in contrast to mRNA, must first enter the nucleus before transcription into mRNA. DNA is consequently less efficient than mRNA because its functionality is contingent on the disintegration of the nuclear envelope during cell division. (2) Unlike viral and DNA vectors, mRNA expresses the encoding proteins transiently and does not insert itself into the genome. As a consequence of its minimal likelihood of insertional mutagenesis, it presents pharmaceutical companies and researchers with an exceptional safety profile. (3) mRNA synthesis via IVT is straightforward. The procedure is reasonably priced and can be implemented expeditiously across various therapeutic modalities. (4) Furthermore, in theory, mRNA has the potential to encode any protein and can therefore be utilized to treat virtually any ailment [141].

In addition, mRNA has the following benefits over alternative RNA-based HPV-16 vaccines: 1) incorporates antigens E6 and E7 into a solitary polypeptide, obviating the need for concurrent administration of two drug products. 2) administers the vaccine via intramuscular injection, thereby ensuring patient compliance and willingness to receive one or more doses; 3) demonstrates preclinical efficacy at a single and modest dose of 3 μg without exhibiting self-amplification characteristics [188].

In addition to their many benefits and conveniences over conventional vaccines, these have several drawbacks. mRNA vaccines are effective against bacterial pathogens and viral pandemics [189]. Assuming the mRNA vaccine to be clinically safe and efficacious confers numerous benefits. One of the primary benefits is the rate of production. As a result of the synthetic output, neither ova nor cells are necessary for this procedure. By manufacturing vaccines within weeks of discovering the genetic sequence of an immunogen, clinical groups can be formed. mRNA technology enables the rapid synthesis of vaccines targeting multiple targets and facilitates the expression of complex proteins that are otherwise challenging or unfeasible to produce [15, 18]. RNA vaccines are non-contagious and devoid of pathogen mutation risk because they do not contain pathogen particles or inactivated pathogens. Further complicating the administration of the vaccine to cells is the rapid degradation of free RNA within the body. mRNA can induce an inflammatory response due to its rapid disruption following injection. Researchers have discovered that the longevity of mRNA can be extended by encapsulating it with LNPs, which are minute droplets of oil [190]. It can serve as an indicator of an inflammatory response to mRNA while imparting transmission. To mitigate this potential hazard, messenger ribonucleic acid vaccines are encapsulated within larger molecules or encapsulated in particles or liposomes, which aid in the stabilization of the RNA chain. A further drawback of mRNA vaccines is the rarity of adverse effects. Vaccine messenger ribonucleic acid chains induce an unintended immune response because foreign sequences designed by body cells resemble those generated by mammalian cells [191].

Nonetheless, the effective conversion of these molecules into pharmaceuticals is hindered by the following obstacles: (i) the enormous size of mRNA; (ii) its inherent instability and susceptibility to degradation by nucleases; and (iii) its activation of the immune system. While chemical modification of mRNA has helped to address a portion of these obstacles, intracellular delivery of mRNA remains a significant barrier. For mRNA-based therapeutics to be translated into clinical practice, delivery technologies that guarantee mRNA stability under physiological conditions are necessary [192].

Particularly during pandemics, their high potency, low cost, and simplicity of production make them optimal candidates for the prevention and treatment of infectious diseases. The obstacles encountered during in vitro RNA transcription were mitigated to some extent through the utilization of tethered adjuvants or the concurrent delivery of unbound mRNA and adjuvant-tethered RNA [193]. By their generation in a cell-free environment via IVT, mRNA vaccines do not entail any inherent risks [194].

Furthermore, it has been demonstrated that chemical modifications to the mRNA molecule, including altered nucleosides and cap structures, are indispensable for addressing concerns related to immunogenicity, attaining long-lasting stability, and facilitating precise and efficient protein synthesis in vivo [195]. Given its minimal adverse-effect profile and rapid manufacturing process, it emerged as an exceptionally promising candidate for a vaccine [196]. Nevertheless, it is important to note that not everything that appears to be gold is gold, as demonstrated by the vaccine recipients' reports of severe reactogenicity and a variety of systemic side effects. These adverse effects are causing a change in attitudes towards the vaccine and contributing to vaccine hesitancy [197]. Although they are uncommon, anaphylaxis, antibody-dependent enhancements, and fatalities are the most severe adverse effects. Temperature sensitivity during storage and transport renders it virtually inaccessible to a nation such as India [198].

mRNA-based therapeutics encompass a broad spectrum of applications, including gene modification, protein replacement therapy, and vaccination. The fact that dozens of mRNA-based vaccine candidates are presently undergoing pre-clinical and clinical development demonstrates that mRNA-based vaccine technology holds great promise as a means to create novel prophylactic and therapeutic vaccines against HPV and malignancies associated with HPV. Translation of mRNA-based therapeutics from the laboratory to the bedside is, nevertheless, impeded by a multitude of challenges arising from the enormous size, charge, intrinsic instability, and high vulnerability to enzymatic degradation of mRNA. Hence, the limited applicability of mRNA-based therapeutics persists due to the requirement for enhanced vectors or drug delivery systems. By utilizing sophisticated delivery systems, one can circumvent the inadequate stability, cell specificity, and translational efficacy exhibited by unmodified mRNA. Nonetheless, numerous mRNA vaccine candidates that have undergone clinical testing are formulated devoid of any delivery system. This indicates that additional advancements in delivery systems for mRNA vaccines are necessary. mRNA delivery presently involves lipoplexes and lipid-based nanoparticles preponderantly. In addition, lipid-polymer hybrid nanoparticles and polymers exhibit considerable potential for cost-effectiveness, safety, stability, and transfection efficiency. Prolonged progress in the formulation and delivery of mRNA via diverse nanomaterials has the potential to enhance the applicability of mRNA in the prevention and treatment of HPV and malignancies associated with HPV [192, 199].

**6. Conclusions**

Although HPV is the most prevalent sexually transmitted disease, there is currently no effective treatment for those who are already infected, and all available vaccines are designed to prevent the disease. Considering the current global population of 200 million individuals infected with HPV, it is evident that the development of a vaccine capable of eradicating the infection and treating the disease is of the utmost importance. In essence, forthcoming prospects for therapeutic vaccines against HPV encompass the refinement of preclinical models, the creation of novel antigen targets, and the development of more potent adjuvants. mRNA vaccine technologies have represented a substantial progression within the domain of vaccine development. Furthermore, the current SARS-CoV-2 pandemic has brought attention to the rapidity and effectiveness with which mRNA vaccines can be manufactured in response to a newly emerging threat. It was recently demonstrated that self-amplifying (sa) RNA formulated with LNP and containing E7 can also inhibit HPV-associated malignancies in rodents. An instance of this is the immunogenicity of mHTV in nonhuman primates, which presents compelling proof that it can be utilized clinically to combat malignancies caused by HPV in humans. Based on all available data, mHTV exhibits potential as a prophylactic and therapeutic vaccine. On the contrary, although vaccines designed to combat infectious diseases are typically administered prophylactically against clearly defined antigens, the majority of anti-tumor vaccines are not administered until the tumor has advanced.

Furthermore, it is worth noting that cancer target antigens are poorly characterized, contain a restricted quantity of cancer-specific cell surface antigens, and exhibit significant interindividual heterogeneity. Moreover, although mRNA vaccines against viruses and malignancies have numerous benefits, they are still in their infancy. Safety is the most important concern at this time. Consideration should be given to antibody-dependent enhancement when designing mRNA vaccines. Their intended application would be evaluated using a cost-benefit ratio. In summary, the character and strength of adaptive immune responses can be influenced by the modulation of the innate immune response. It is essential to investigate the early interaction between the innate and adaptive immune systems to attain an optimal, protracted, and enhanced immune response, as well as to protect effectively and restrict inflammation. mRNA vaccines have demonstrated limited efficacy in the clinical phase despite their overall effectiveness in preclinical investigations. Further developments in the future will involve the investigation of additional in situ tumor models, the implementation of combination medication therapy, and the development of novel antigenic targets (e.g., E1 and E5) to enhance the effectiveness of the mRNA vaccine. We maintain optimism regarding the potential of mRNA vaccines to treat and prevent HPV effectively.

**Ethics approval and consent to participate**

Not applicable.

**Consent for publication**

All author consent for publication

**Availability of data and material**

Not applicable.

**Competing interests**

The authors declare that they have no competing interests

**Funding**

Not applicable.

**Authors' contributions**

S.Y., and O.G., Writing – original draft. F.M., S.D., P.M., M.S.M., O.Q., H.N.S., M.A., H.Z., reviewed and edited. All authors read and approved the final manuscript.

**Acknowledgements**

Special thanks to all scientists in the field of obstetrics and gynecology

**References:**

1. Yousefi, Z., et al., *An update on human papilloma virus vaccines: history, types, protection, and efficacy.* Frontiers in Immunology, 2022. **12**: p. 805695.

2. Bonnez, W., *Human Papillomavirus.* Vaccines for Biodefense and Emerging and Neglected Diseases; Alan, DT, Barrett, LRS, Eds, 2009: p. 469-496.

3. Zur Hausen, H. and E.-M. De Villiers, *Human papillomaviruses.* Annual review of microbiology, 1994. **48**(1): p. 427-447.

4. Karamanou, M., et al., *From the humble wart to HPV: a fascinating story throughout centuries.* Oncology Reviews, 2010. **4**: p. 133-135.

5. Tirosh, O., et al., *Expanded skin virome in DOCK8-deficient patients.* Nature medicine, 2018. **24**(12): p. 1815-1821.

6. Chen, Z., et al., *Niche adaptation and viral transmission of human papillomaviruses from archaic hominins to modern humans.* PLoS pathogens, 2018. **14**(11): p. e1007352.

7. Rodríguez-Álvarez, M.I., et al., *Prevalence and risk factors of human papillomavirus in male patients: a systematic review and meta-analysis.* International journal of environmental research and public health, 2018. **15**(10): p. 2210.

8. Luhn, P., et al., *The role of co-factors in the progression from human papillomavirus infection to cervical cancer.* Gynecologic oncology, 2013. **128**(2): p. 265-270.

9. Jain, M., et al., *Epidemiology, Molecular Pathogenesis, Immuno-Pathogenesis, Immune Escape Mechanisms and Vaccine Evaluation for HPV-Associated Carcinogenesis.* Pathogens, 2023. **12**(12): p. 1380.

10. Almeida, A.M., et al., *Cervical cancer and HPV infection: ongoing therapeutic research to counteract the action of E6 and E7 oncoproteins.* Drug discovery today, 2019. **24**(10): p. 2044-2057.

11. Taghizadeh, E., et al., *Roles of E6 and E7 human papillomavirus proteins in molecular pathogenesis of cervical cancer.* Current Protein and Peptide Science, 2019. **20**(9): p. 926-934.

12. Smalley Rumfield, C., et al., *Therapeutic vaccines for HPV-associated malignancies.* ImmunoTargets and therapy, 2020: p. 167-200.

13. Aggarwal, S., P. Agarwal, and N. Gupta, *A comprehensive narrative review of challenges and facilitators in the implementation of various HPV vaccination program worldwide.* Cancer Medicine, 2024.

14. Ghebreyesus, T., *WHO Director-General calls for all countries to take action to help end the suffering caused by cervical cancer*. 2018, Geneva: WHO, 2018. https://www. who. int/reproductivehealth/call-to-action ….

15. Rauch, S., et al., *New vaccine technologies to combat outbreak situations.* Frontiers in immunology, 2018. **9**: p. 1963.

16. Pollard, A.J. and E.M. Bijker, *A guide to vaccinology: from basic principles to new developments.* Nature Reviews Immunology, 2021. **21**(2): p. 83-100.

17. Leppek, K., R. Das, and M. Barna, *Functional 5′ UTR mRNA structures in eukaryotic translation regulation and how to find them.* Nature reviews Molecular cell biology, 2018. **19**(3): p. 158-174.

18. Maruggi, G., et al., *mRNA as a transformative technology for vaccine development to control infectious diseases.* Molecular Therapy, 2019. **27**(4): p. 757-772.

19. Jackson, N.A., et al., *The promise of mRNA vaccines: a biotech and industrial perspective.* npj Vaccines, 2020. **5**(1): p. 11.

20. Chahal, J.S., et al., *An RNA nanoparticle vaccine against Zika virus elicits antibody and CD8+ T cell responses in a mouse model.* Scientific reports, 2017. **7**(1): p. 252.

21. De Martel, C., et al., *Worldwide burden of cancer attributable to HPV by site, country and HPV type.* International journal of cancer, 2017. **141**(4): p. 664-670.

22. Kowalzik, F., et al., *mRNA-based vaccines.* Vaccines, 2021. **9**(4): p. 390.

23. Zhang, G., et al., *mRNA vaccines in disease prevention and treatment.* Signal Transduction and Targeted Therapy, 2023. **8**(1): p. 365.

24. Rzymski, P., et al., *mRNA vaccines: The future of prevention of viral infections?* J Med Virol, 2023. **95**(2): p. e28572.

25. Whitaker, J.A., H.M.E. Sahly, and C.M. Healy, *mRNA vaccines against respiratory viruses.* Curr Opin Infect Dis, 2023. **36**(5): p. 385-393.

26. Saadh, M.J., et al., *Progress and prospects on vaccine development against monkeypox infection.* Microbial Pathogenesis, 2023: p. 106156.

27. Nasiri, K., et al., *Spotlight on the impact of viral infections on Hematopoietic Stem Cells (HSCs) with a focus on COVID-19 effects.* Cell Communication and Signaling, 2023. **21**(1): p. 103.

28. Barbier, A.J., et al., *The clinical progress of mRNA vaccines and immunotherapies.* Nature biotechnology, 2022. **40**(6): p. 840-854.

29. Macartney, K.K., et al., *Safety of human papillomavirus vaccines: a review.* Drug safety, 2013. **36**: p. 393-412.

30. Firdaus, M.E.R., et al., *Optimization, characterization, comparison of self-assembly VLP of capsid protein L1 in yeast and reverse vaccinology design against human papillomavirus type 52.* Journal of Genetic Engineering and Biotechnology, 2023. **21**(1): p. 1-13.

31. de Moura, I.A. and A.J.D. Silva, *Enhancing the Effect of Nucleic Acid Vaccines in the Treatment of HPV-Related Cancers: An Overview of Delivery Systems.* 2022. **11**(12).

32. Mo, Y., et al., *Prophylactic and therapeutic HPV vaccines: current scenario and perspectives.* Frontiers in Cellular and Infection Microbiology, 2022. **12**: p. 909223.

33. Cantrell, S.C., et al., *Differences in imaging characteristics of HPV-positive and HPV-negative oropharyngeal cancers: a blinded matched-pair analysis.* American Journal of Neuroradiology, 2013. **34**(10): p. 2005-2009.

34. Chen, Z., et al., *Characteristics of HPV prevalence in Sichuan Province, China.* International Journal of Gynecology & Obstetrics, 2015. **131**(3): p. 277-280.

35. Rakislova, N., et al., *Histological characteristics of HPV‐associated and‐independent squamous cell carcinomas of the vulva: a study of 1,594 cases.* International journal of cancer, 2017. **141**(12): p. 2517-2527.

36. Bosch, F.X. and S.d. Sanjosé, *Human papillomavirus in cervical cancer.* Current oncology reports, 2002. **4**: p. 175-184.

37. Bosch, F.X., et al., *The causal relation between human papillomavirus and cervical cancer.* Journal of clinical pathology, 2002. **55**(4): p. 244.

38. Wang, Y., et al., *Characteristics of human papillomavirus infection among females and the genetic variations of HPV18 and HPV58 in Henan province, China.* Scientific Reports, 2023. **13**(1): p. 2252.

39. Woods, M. and P.A. Reichart, *87-Surgical Management of Nonmalignant Lesions of the Mouth, Editor (s): Peter A.* Brennan, Henning Schliephake, GE Ghali, Luke Cascarini, Maxillofacial Surgery (Third Edition), Churchill Livingstone, 2017: p. 1319-1334.

40. Knight, G.L., et al., *A cyclin-binding motif in human papillomavirus type 18 (HPV18) E1^ E4 is necessary for association with CDK–cyclin complexes and G2/M cell cycle arrest of keratinocytes, but is not required for differentiation-dependent viral genome amplification or L1 capsid protein expression.* Virology, 2011. **412**(1): p. 196-210.

41. Gholizadeh, O., et al., *Review of the evidence of the effects of human papillomavirus infection and Gardnerella vaginalis, and their co-infection on infertility.* Microbial Pathogenesis, 2023. **175**: p. 105980.

42. Woodby, B., M. Scott, and J. Bodily, *The interaction between human papillomaviruses and the stromal microenvironment.* Progress in molecular biology and translational science, 2016. **144**: p. 169-238.

43. Bzhalava, D., C. Eklund, and J. Dillner, *International standardization and classification of human papillomavirus types.* Virology, 2015. **476**: p. 341-344.

44. de Villiers, E.-M., *Cross-roads in the classification of papillomaviruses.* Virology, 2013. **445**(1-2): p. 2-10.

45. Dona, M.G., et al., *Alpha, beta and gamma Human Papillomaviruses in the anal canal of HIV-infected and uninfected men who have sex with men.* Journal of Infection, 2015. **71**(1): p. 74-84.

46. Mlakar, B., et al., *Betapapillomaviruses in the anal canal of HIV positive and HIV negative men who have sex with men.* Journal of Clinical Virology, 2014. **61**(2): p. 237-241.

47. Smelov, V., et al., *Beta and gamma human papillomaviruses in anal and genital sites among men: prevalence and determinants.* Scientific reports, 2018. **8**(1): p. 8241.

48. Merino, S., et al., *Mechanisms of Klebsiella pneumoniae resistance to complement-mediated killing.* Infection and immunity, 1992. **60**(6): p. 2529-2535.

49. Johnson, D.E., et al., *Head and neck squamous cell carcinoma.* Nature reviews Disease primers, 2020. **6**(1): p. 92.

50. Giuliani, E., et al., *Human Papillomavirus Oral Infection: Review of Methodological Aspects and Epidemiology.* Pathogens, 2021. **10**(11): p. 1411.

51. Nelson, C.W. and L. Mirabello, *Human papillomavirus genomics: Understanding carcinogenicity.* Tumour Virus Research, 2023. **15**: p. 200258.

52. Yu, L., V. Majerciak, and Z.-M. Zheng, *HPV16 and HPV18 genome structure, expression, and post-transcriptional regulation.* International journal of molecular sciences, 2022. **23**(9): p. 4943.

53. Li, N., et al., *Structure of the origin recognition complex bound to DNA replication origin.* Nature, 2018. **559**(7713): p. 217-222.

54. Zhu, Y., et al., *Human papillomavirus and its testing assays, cervical cancer screening, and vaccination.* Advances in clinical chemistry, 2017. **81**: p. 135-192.

55. Alemany, L., et al., *Role of human papillomavirus in penile carcinomas worldwide.* European urology, 2016. **69**(5): p. 953-961.

56. Vorsters, A., et al., *HPV prevention and control–the way forward.* Preventive medicine, 2022. **156**: p. 106960.

57. Stevenson, A., et al., *Risk stratification of cervical disease using detection of human papillomavirus (HPV) E4 protein and cellular MCM protein in clinical liquid based cytology samples.* Journal of Clinical Virology, 2018. **108**: p. 19-25.

58. MAGAJI, Y.G., et al., *An overview of the general characteristics, pathogenicity, transmission, and diagnosis of human papilloma virus (HPV).* Gadau Journal of Pure and Allied Sciences, 2022. **1**(1): p. 27-39.

59. Antonishyn, N.A., *The utility of HPV typing and relative quantification of HPV-16 transcripts for monitoring HPV vaccine efficacy and improving colposcopy triage of women with abnormal cervical cytology*. 2007, Faculty of Graduate Studies and Research, University of Regina.

60. D'Abramo, C.M. and J. Archambault, *Small molecule inhibitors of human papillomavirus protein - protein interactions.* Open Virol J, 2011. **5**: p. 80-95.

61. Van Doorslaer, K., et al., *The Papillomavirus Episteme: a major update to the papillomavirus sequence database.* Nucleic acids research, 2017. **45**(D1): p. D499-D506.

62. Cobo, F., *Human papillomavirus infections: From the laboratory to clinical practice*. 2012: Elsevier.

63. Bruni, L., et al., *Human papillomavirus and related diseases in the world.* Summary report, 2019. **17**.

64. Forman, D., et al., *Global burden of human papillomavirus and related diseases.* Vaccine, 2012. **30**: p. F12-F23.

65. Bruni, L., et al., *Cervical human papillomavirus prevalence in 5 continents: meta-analysis of 1 million women with normal cytological findings.* Journal of Infectious Diseases, 2010. **202**(12): p. 1789-1799.

66. Alizon, S., C.L. Murall, and I.G. Bravo, *Why human papillomavirus acute infections matter.* Viruses, 2017. **9**(10): p. 293.

67. Tan, S.C., et al., *Prevalence and type distribution of human papillomavirus (HPV) in Malaysian women with and without cervical cancer: an updated estimate.* Bioscience reports, 2018. **38**(2): p. BSR20171268.

68. Cogliano, V., et al., *Carcinogenicity of human papillomaviruses.* The lancet oncology, 2005. **6**(4): p. 204.

69. Franceschi, S., et al., *Variations in the age‐specific curves of human papillomavirus prevalence in women worldwide.* International journal of cancer, 2006. **119**(11): p. 2677-2684.

70. Arbyn, M., et al., *Estimates of incidence and mortality of cervical cancer in 2018: a worldwide analysis.* The Lancet Global Health, 2020. **8**(2): p. e191-e203.

71. Johnson, C., N. Obanor, and A. DeWeese, *Human papillomavirus and cancer in men.* Health Science Journal, 2016. **10**(6): p. 1.

72. Patel, P., et al., *Prevalence, incidence, and clearance of anal high-risk human papillomavirus infection among HIV-infected men in the SUN study.* The Journal of infectious diseases, 2018. **217**(6): p. 953-963.

73. Moreira Jr, E.D., et al., *Incidence, clearance, and disease progression of genital human papillomavirus infection in heterosexual men.* The Journal of infectious diseases, 2014. **210**(2): p. 192-199.

74. Giuliano, A.R., et al., *The human papillomavirus infection in men study: human papillomavirus prevalence and type distribution among men residing in Brazil, Mexico, and the United States.* Cancer Epidemiology Biomarkers & Prevention, 2008. **17**(8): p. 2036-2043.

75. Sung, H., et al., *Global cancer statistics 2020: GLOBOCAN estimates of incidence and mortality worldwide for 36 cancers in 185 countries.* CA: a cancer journal for clinicians, 2021. **71**(3): p. 209-249.

76. Pesola, F. and P. Sasieni, *Impact of screening on cervical cancer incidence in England: a time trend analysis.* BMJ open, 2019. **9**(1).

77. Mendes, D., et al., *Understanding differences in cervical cancer incidence in Western Europe: comparing Portugal and England.* The European Journal of Public Health, 2018. **28**(2): p. 343-347.

78. Mesher, D., et al., *Type-specific HPV prevalence in invasive cervical cancer in the UK prior to national HPV immunisation programme: baseline for monitoring the effects of immunisation.* Journal of Clinical Pathology, 2014.

79. Wang, X., X. Huang, and Y. Zhang, *Involvement of human papillomaviruses in cervical cancer.* Frontiers in microbiology, 2018. **9**: p. 2896.

80. Petca, A., et al., *Non-sexual HPV transmission and role of vaccination for a better future.* Experimental and therapeutic medicine, 2020. **20**(6): p. 1-1.

81. Choi, S., et al., *HPV and cervical cancer: A review of epidemiology and screening uptake in the UK.* Pathogens, 2023. **12**(2): p. 298.

82. Scott-Wittenborn, N. and C. Fakhry. *Epidemiology of HPV related malignancies*. in *Seminars in Radiation Oncology*. 2021. Elsevier.

83. Lu, Y., et al., *Global burden of oropharyngeal cancer attributable to human papillomavirus by anatomical subsite and geographic region.* Cancer Epidemiology, 2022. **78**: p. 102140.

84. Rettig, E.M., et al., *Oropharyngeal cancer is no longer a disease of younger patients and the prognostic advantage of Human Papillomavirus is attenuated among older patients: Analysis of the National Cancer Database.* Oral oncology, 2018. **83**: p. 147-153.

85. Ndon, S., et al., *Human papillomavirus-associated oropharyngeal cancer: global epidemiology and public policy implications.* Cancers, 2023. **15**(16): p. 4080.

86. Yang, X., et al., *Cervical HPV infection in Guangzhou, China: an epidemiological study of 198,111 women from 2015 to 2021.* Emerging Microbes & Infections, 2023. **12**(1): p. e2176009.

87. de Martel, C., et al., *Global burden of cancer attributable to infections in 2018: a worldwide incidence analysis.* The Lancet Global Health, 2020. **8**(2): p. e180-e190.

88. Sasidharanpillai, S., et al., *Prevalence of human papillomavirus (HPV) DNA among men with oropharyngeal and anogenital cancers: a systematic review and meta-analysis.* Asian Pacific journal of cancer prevention: APJCP, 2021. **22**(5): p. 1351.

89. Carlander, A.F., et al., *A contemporary systematic review on repartition of HPV-positivity in oropharyngeal cancer worldwide.* Viruses, 2021. **13**(7): p. 1326.

90. Gabutti, G., et al., *Human papilloma virus vaccination: Focus on the italian situation.* Vaccines, 2021. **9**(12): p. 1374.

91. Seedat, R., *Juvenile-onset recurrent respiratory papillomatosis diagnosis and management–a developing country review.* Pediatric health, medicine and therapeutics, 2020: p. 39-46.

92. Organista‑Nava, J., et al., *Cervical cancer stem cell‑associated genes: Prognostic implications in cervical cancer.* Oncology Letters, 2019. **18**(1): p. 7-14.

93. Ramachandran, D. and T. Dörk, *Genomic risk factors for cervical cancer.* Cancers, 2021. **13**(20): p. 5137.

94. Faghihkhorasani, A., et al., *The role of oncolytic virotherapy and viral oncogenes in the cancer stem cells: a review of virus in cancer stem cells.* Cancer Cell International, 2023. **23**(1): p. 250.

95. Drolet, M., et al., *Population-level impact and herd effects following the introduction of human papillomavirus vaccination programmes: updated systematic review and meta-analysis.* The Lancet, 2019. **394**(10197): p. 497-509.

96. Setiawan, D., et al., *The clinical effectiveness of one-dose vaccination with an HPV vaccine: A meta-analysis of 902,368 vaccinated women.* Plos one, 2024. **19**(1): p. e0290808.

97. Schiller, J.T., X. Castellsagué, and S.M. Garland, *A review of clinical trials of human papillomavirus prophylactic vaccines.* Vaccine, 2012. **30**: p. F123-F138.

98. Skolnik, J.M. and M.P. Morrow, *Vaccines for HPV-associated diseases.* Molecular Aspects of Medicine, 2023. **94**: p. 101224.

99. Brisson, M., et al., *Impact of HPV vaccination and cervical screening on cervical cancer elimination: a comparative modelling analysis in 78 low-income and lower-middle-income countries.* The Lancet, 2020. **395**(10224): p. 575-590.

100. Gupta, G., R. Glueck, and P.R. Patel, *HPV vaccines: Global perspectives.* Human vaccines & immunotherapeutics, 2017. **13**(6): p. 1421-1424.

101. Wang, R., et al., *Human papillomavirus vaccine against cervical cancer: Opportunity and challenge.* Cancer letters, 2020. **471**: p. 88-102.

102. Pils, S. and E. Joura, *From the monovalent to the nine-valent HPV vaccine.* Clinical Microbiology and Infection, 2015. **21**(9): p. 827-833.

103. Hu, Y., et al., *Safety of an Escherichia coli-expressed bivalent human papillomavirus (types 16 and 18) L1 virus-like particle vaccine: an open-label phase I clinical trial.* Human vaccines & immunotherapeutics, 2014. **10**(2): p. 469-475.

104. Bryan, J.T., *Developing an HPV vaccine to prevent cervical cancer and genital warts.* Vaccine, 2007. **25**(16): p. 3001-3006.

105. Illah, O. and A. Olaitan, *Updates on HPV Vaccination.* Diagnostics, 2023. **13**(2): p. 243.

106. Huh, W.K., et al., *Final efficacy, immunogenicity, and safety analyses of a nine-valent human papillomavirus vaccine in women aged 16–26 years: a randomised, double-blind trial.* The Lancet, 2017. **390**(10108): p. 2143-2159.

107. Guevara, A., et al., *Antibody persistence and evidence of immune memory at 5 years following administration of the 9-valent HPV vaccine.* Vaccine, 2017. **35**(37): p. 5050-5057.

108. Huber, J., et al., *Human papillomavirus persistence or clearance after infection in reproductive age. What is the status? Review of the literature and new data of a vaginal gel containing silicate dioxide, citric acid, and selenite.* Women's Health, 2021. **17**: p. 17455065211020702.

109. Skinner, S.R., et al., *Efficacy, safety, and immunogenicity of the human papillomavirus 16/18 AS04-adjuvanted vaccine in women older than 25 years: 4-year interim follow-up of the phase 3, double-blind, randomised controlled VIVIANE study.* The Lancet, 2014. **384**(9961): p. 2213-2227.

110. Donken, R., et al., *Immunogenicity of 2 and 3 doses of the quadrivalent human papillomavirus vaccine up to 120 months postvaccination: follow-up of a randomized clinical trial.* Clinical Infectious Diseases, 2020. **71**(4): p. 1022-1029.

111. Markowitz, L.E. and J.T. Schiller, *Human papillomavirus vaccines.* The Journal of infectious diseases, 2021. **224**(Supplement_4): p. S367-S378.

112. Qiao, Y.-L., et al., *Efficacy, safety, and immunogenicity of an Escherichia coli-produced bivalent human papillomavirus vaccine: an interim analysis of a randomized clinical trial.* JNCI: Journal of the National Cancer Institute, 2020. **112**(2): p. 145-153.

113. Reuschenbach, M., et al., *Prophylactic HPV vaccines in patients with HPV-associated diseases and cancer.* Vaccine, 2023.

114. Gilca, V., et al., *Immunogenicity and safety of a mixed vaccination schedule with one dose of nonavalent and one dose of bivalent HPV vaccine versus two doses of nonavalent vaccine–A randomized clinical trial.* Vaccine, 2018. **36**(46): p. 7017-7024.

115. Lyseng-Williamson, K.A., *Human papillomavirus-16/18 AS04-adjuvanted vaccine (cervarix®): a guide to its two-dose schedule in girls aged 9–14 years in the EU.* Pediatric Drugs, 2014. **16**(3): p. 247-253.

116. Boxus, M., et al., *Antibody avidity measurements in recipients of Cervarix® vaccine following a two-dose schedule or a three-dose schedule.* Vaccine, 2014. **32**(26): p. 3232-3236.

117. Wang, W., et al., *Real-world impact and effectiveness of the quadrivalent HPV vaccine: an updated systematic literature review.* Expert Review of Vaccines, 2022. **21**(12): p. 1799-1817.

118. Meites, E., *Use of a 2-dose schedule for human papillomavirus vaccination—updated recommendations of the Advisory Committee on Immunization Practices.* MMWR. Morbidity and mortality weekly report, 2016. **65**.

119. Soliman, M., O. Oredein, and C.R. Dass, *Update on safety and efficacy of HPV vaccines: focus on gardasil.* International Journal of Molecular and Cellular Medicine, 2021. **10**(2): p. 101.

120. Cuzick, J., *Gardasil 9 joins the fight against cervix cancer.* Expert review of vaccines, 2015. **14**(8): p. 1047-1049.

121. Printz, C., *FDA approves Gardasil 9 for more types of HPV.* Cancer, 2015. **121**(8): p. 1156-1157.

122. Elliott, W. and J. Chan, *Human Papillomavirus Vaccine (Gardasil 9).* Internal Medicine Alert, 2018. **40**(21).

123. Pasmans, H., et al., *Characterization of the early cellular immune response induced by HPV vaccines.* Frontiers in Immunology, 2022. **13**: p. 863164.

124. Garbuglia, A.R., et al., *The use of both therapeutic and prophylactic vaccines in the therapy of papillomavirus disease.* Frontiers in immunology, 2020. **11**: p. 188.

125. Romanowski, B., et al., *Sustained efficacy and immunogenicity of the human papillomavirus (HPV)-16/18 AS04-adjuvanted vaccine: analysis of a randomised placebo-controlled trial up to 6.4 years.* Lancet (London, England), 2009. **374**(9706): p. 1975-1985.

126. Pinto, L.A., et al., *Immunogenicity of HPV prophylactic vaccines: Serology assays and their use in HPV vaccine evaluation and development.* Vaccine, 2018. **36**(32 Pt A): p. 4792-4799.

127. Einstein, M.H., et al., *Comparison of long-term immunogenicity and safety of human papillomavirus (HPV)-16/18 AS04-adjuvanted vaccine and HPV-6/11/16/18 vaccine in healthy women aged 18-45 years: end-of-study analysis of a Phase III randomized trial.* Human vaccines & immunotherapeutics, 2014. **10**(12): p. 3435-3445.

128. Einstein, M.H., et al., *Comparative immunogenicity and safety of human papillomavirus (HPV)-16/18 vaccine and HPV-6/11/16/18 vaccine: follow-up from months 12–24 in a Phase III randomized study of healthy women aged 18–45 years.* Human vaccines, 2011. **7**(12): p. 1343-1358.

129. Malone, R.W., P.L. Felgner, and I.M. Verma, *Cationic liposome-mediated RNA transfection.* Proceedings of the National Academy of Sciences, 1989. **86**(16): p. 6077-6081.

130. Markowitz, L.E., et al., *Prevalence of HPV after introduction of the vaccination program in the United States.* Pediatrics, 2016. **137**(3).

131. Thess, A., et al., *Sequence-engineered mRNA without chemical nucleoside modifications enables an effective protein therapy in large animals.* Molecular Therapy, 2015. **23**(9): p. 1456-1464.

132. Pardi, N., et al., *mRNA vaccines—a new era in vaccinology.* Nature reviews Drug discovery, 2018. **17**(4): p. 261-279.

133. Pollard, C., et al., *Challenges and advances towards the rational design of mRNA vaccines.* Trends in molecular medicine, 2013. **19**(12): p. 705-713.

134. Sullenger, B.A. and S. Nair, *From the RNA world to the clinic.* Science, 2016. **352**(6292): p. 1417-1420.

135. Steinle, H., et al., *Concise review: application of in vitro transcribed messenger RNA for cellular engineering and reprogramming: progress and challenges.* Stem Cells, 2017. **35**(1): p. 68-79.

136. Armbruster, N., E. Jasny, and B. Petsch, *Advances in RNA vaccines for preventive indications: a case study of a vaccine against rabies.* Vaccines, 2019. **7**(4): p. 132.

137. Granados-Riveron, J.T. and G. Aquino-Jarquin, *Engineering of the current nucleoside-modified mRNA-LNP vaccines against SARS-CoV-2.* Biomedicine & Pharmacotherapy, 2021. **142**: p. 111953.

138. Jackson, L.A., et al., *An mRNA vaccine against SARS-CoV-2—preliminary report.* New England journal of medicine, 2020. **383**(20): p. 1920-1931.

139. Verbeke, R., et al., *The dawn of mRNA vaccines: The COVID-19 case.* Journal of Controlled Release, 2021. **333**: p. 511-520.

140. Kim, S.C., et al., *Modifications of mRNA vaccine structural elements for improving mRNA stability and translation efficiency.* Molecular & Cellular Toxicology, 2022: p. 1-8.

141. Weng, Y., et al., *The challenge and prospect of mRNA therapeutics landscape.* Biotechnology advances, 2020. **40**: p. 107534.

142. Diken, M., et al., *Selective uptake of naked vaccine RNA by dendritic cells is driven by macropinocytosis and abrogated upon DC maturation.* Gene therapy, 2011. **18**(7): p. 702-708.

143. Al Fayez, N., et al., *Recent Advancement in mRNA Vaccine Development and Applications.* Pharmaceutics, 2023. **15**(7): p. 1972.

144. Yasamineh, S., et al., *An overview on nanoparticle-based strategies to fight viral infections with a focus on COVID-19.* Journal of Nanobiotechnology, 2022. **20**(1): p. 1-26.

145. Yasamineh, S., et al., *A state-of-the-art review on the recent advances of niosomes as a targeted drug delivery system.* International journal of pharmaceutics, 2022. **624**: p. 121878.

146. Zhou, K., et al., *Modular degradable dendrimers enable small RNAs to extend survival in an aggressive liver cancer model.* Proceedings of the National Academy of Sciences, 2016. **113**(3): p. 520-525.

147. Ulkoski, D., et al., *Recent advances in polymeric materials for the delivery of RNA therapeutics.* Expert Opinion on Drug Delivery, 2019. **16**(11): p. 1149-1167.

148. Kim, Y., et al., *The potential of cell-penetrating peptides for mRNA delivery to cancer cells.* Pharmaceutics, 2022. **14**(6): p. 1271.

149. Linares-Fernández, S., et al., *Tailoring mRNA vaccine to balance innate/adaptive immune response.* Trends in molecular medicine, 2020. **26**(3): p. 311-323.

150. Rosa, S.S., et al., *mRNA vaccines manufacturing: Challenges and bottlenecks.* Vaccine, 2021. **39**(16): p. 2190-2200.

151. Gunter, H.M., et al., *mRNA vaccine quality analysis using RNA sequencing.* Nature Communications, 2023. **14**(1): p. 5663.

152. Kowalski, P.S., et al., *Delivering the messenger: advances in technologies for therapeutic mRNA delivery.* Molecular Therapy, 2019. **27**(4): p. 710-728.

153. Pardi, N., M.J. Hogan, and D. Weissman, *Recent advances in mRNA vaccine technology.* Current opinion in immunology, 2020. **65**: p. 14-20.

154. Zhao, Y., et al., *Multiple injections of electroporated autologous T cells expressing a chimeric antigen receptor mediate regression of human disseminated tumor.* Cancer research, 2010. **70**(22): p. 9053-9061.

155. Rydzik, A.M., et al., *Synthesis and properties of mRNA cap analogs containing imidodiphosphate moiety—Fairly mimicking natural cap structure, yet resistant to enzymatic hydrolysis.* Bioorganic & medicinal chemistry, 2012. **20**(5): p. 1699-1710.

156. Schlake, T., et al., *Developing mRNA-vaccine technologies.* RNA biology, 2012. **9**(11): p. 1319-1330.

157. Maassen, S.J., P. van der Schoot, and J.J. Cornelissen, *Experimental and Theoretical Determination of the pH inside the Confinement of a Virus‐Like Particle.* Small, 2018. **14**(36): p. 1802081.

158. Crone, M.A., et al., *A role for Biofoundries in rapid development and validation of automated SARS-CoV-2 clinical diagnostics.* Nature communications, 2020. **11**(1): p. 4464.

159. Wang, W., et al., *Dual-targeting nanoparticle vaccine elicits a therapeutic antibody response against chronic hepatitis B.* Nature nanotechnology, 2020. **15**(5): p. 406-416.

160. Jarzebska, N.T., et al., *Protamine-based strategies for RNA transfection.* Pharmaceutics, 2021. **13**(6): p. 877.

161. Ruseska, I., et al., *Use of protamine in nanopharmaceuticals—A review.* Nanomaterials, 2021. **11**(6): p. 1508.

162. Hou, X., et al., *Lipid nanoparticles for mRNA delivery.* Nature Reviews Materials, 2021. **6**(12): p. 1078-1094.

163. Patel, S., et al., *Brief update on endocytosis of nanomedicines.* Advanced drug delivery reviews, 2019. **144**: p. 90-111.

164. Veiga, N., et al., *Cell specific delivery of modified mRNA expressing therapeutic proteins to leukocytes.* Nature communications, 2018. **9**(1): p. 4493.

165. Huang, X., et al., *Identification of tumor antigens and immune subtypes of pancreatic adenocarcinoma for mRNA vaccine development.* Molecular Cancer, 2021. **20**: p. 1-18.

166. Ahmad, Z. and C.L. Poh, *The conserved molecular determinants of virulence in dengue virus.* International journal of medical sciences, 2019. **16**(3): p. 355.

167. Faghihkhorasani, A., et al., *The potential use of bacteria and bacterial derivatives as drug delivery systems for viral infection.* Virology Journal, 2023. **20**(1): p. 222.

168. Guevara, M.L., F. Persano, and S. Persano, *Advances in lipid nanoparticles for mRNA-based cancer immunotherapy.* Frontiers in chemistry, 2020. **8**: p. 589959.

169. Rafael, T.S., et al., *Immunotherapeutic approaches for the treatment of HPV-associated (pre-) cancer of the cervix, vulva and penis.* Journal of Clinical Medicine, 2022. **11**(4): p. 1101.

170. Gong, X., et al., *Advances in HPV‐associated tumor management: Therapeutic strategies and emerging insights.* Journal of Medical Virology, 2023. **95**(7): p. e28950.

171. Moch, H., *Female genital tumours: WHO Classification of Tumours, Volume 4.* WHO Classification of Tumours, 2020. **4**.

172. dos Santos Melli, P.P., G. Duarte, and S.M. Quintana, *Multivariate analysis of risk factors for the persistence of high-grade squamous intraepithelial lesions following loop electrosurgical excision procedure.* International Journal of Gynecology & Obstetrics, 2016. **133**(2): p. 234-237.

173. Chen, Y., et al., *A comparative study of treatment of cervical low-grade squamous intraepithelial lesions (LSIL).* Photodiagnosis and Photodynamic Therapy, 2024. **45**: p. 103920.

174. Li, Y., et al., *Effects of 5-aminolevulinic acid photodynamic therapy for cervical low-grade squamous intraepithelial lesions with HR-HPV infections.* Frontiers in Medicine, 2024. **10**: p. 1301440.

175. Perkins, R.B., et al., *2019 ASCCP risk-based management consensus guidelines for abnormal cervical cancer screening tests and cancer precursors.* Journal of lower genital tract disease, 2020. **24**(2): p. 102-131.

176. Ramos da Silva, J., et al., *Single immunizations of self-amplifying or non-replicating mRNA-LNP vaccines control HPV-associated tumors in mice.* Science Translational Medicine, 2023. **15**(686): p. eabn3464.

177. Qiu, K., et al., *mRNA-LNP vaccination-based immunotherapy augments CD8+ T cell responses against HPV-positive oropharyngeal cancer.* npj Vaccines, 2023. **8**(1): p. 144.

178. Lee, S., et al., *mRNA‐HPV vaccine encoding E6 and E7 improves therapeutic potential for HPV‐mediated cancers via subcutaneous immunization.* Journal of Medical Virology, 2023. **95**(12): p. e29309.

179. Zhou, K., et al., *HPV16 E6/E7-based mRNA vaccine is therapeutic in mice bearing aggressive HPV-positive lesions.* Frontiers in Immunology, 2023. **14**.

180. Moser, J., et al., *183P COMMANDER-001: Initial safety data from a phase I/II dose escalation/expansion study of SQZ-eAPC-HPV, a cell-based mRNA therapeutic cancer vaccine for HPV16+ solid tumors.* Immuno-Oncology and Technology, 2022. **16**.

181. Grunwitz, C., et al., *HPV16 RNA-LPX vaccine mediates complete regression of aggressively growing HPV-positive mouse tumors and establishes protective T cell memory.* Oncoimmunology, 2019. **8**(9): p. e1629259.

182. Li, O., et al., *134 mNTX-250, a novel multimodal HPV-16 mRNA-based therapeutic, induces potent anti-tumor responses and establishes HPV-16 specific immune memory*. 2023, BMJ Specialist Journals.

183. Muderspach, L., et al., *A phase I trial of a human papillomavirus (HPV) peptide vaccine for women with high-grade cervical and vulvar intraepithelial neoplasia who are HPV 16 positive.* Clinical Cancer Research, 2000. **6**(9): p. 3406-3416.

184. Pardi, N., et al., *Zika virus protection by a single low-dose nucleoside-modified mRNA vaccination.* Nature, 2017. **543**(7644): p. 248-251.

185. Richner, J.M., et al., *Modified mRNA vaccines protect against Zika virus infection.* Cell, 2017. **168**(6): p. 1114-1125. e10.

186. Lutz, J., et al., *Unmodified mRNA in LNPs constitutes a competitive technology for prophylactic vaccines.* npj Vaccines, 2017. **2**(1): p. 29.

187. Aldrich, C., et al., *Proof-of-concept of a low-dose unmodified mRNA-based rabies vaccine formulated with lipid nanoparticles in human volunteers: A phase 1 trial.* Vaccine, 2021. **39**(8): p. 1310-1318.

188. Zhou, K., et al., *HPV16 E6/E7 -based mRNA vaccine is therapeutic in mice bearing aggressive HPV-positive lesions.* Front Immunol, 2023. **14**: p. 1213285.

189. Mutlu, Z.S., İ. Üzümcü, and O. Erbaş, *mRNA Vaccines.* Journal of Experimental and Basic Medical Sciences, 2021. **2**(2): p. 267-273.

190. Meyer, M., et al., *Modified mRNA-based vaccines elicit robust immune responses and protect guinea pigs from Ebola virus disease.* The Journal of infectious diseases, 2018. **217**(3): p. 451-455.

191. Scorza, F.B. and N. Pardi, *New kids on the block: RNA-based influenza virus vaccines.* Vaccines, 2018. **6**(2): p. 20.

192. Wadhwa, A., et al., *Opportunities and Challenges in the Delivery of mRNA-based Vaccines.* Pharmaceutics, 2020. **12**(2).

193. Karam, M. and G. Daoud, *mRNA vaccines: Past, present, future.* Asian journal of pharmaceutical sciences, 2022.

194. Li, B., X. Luo, and Y. Dong, *Effects of chemically modified messenger RNA on protein expression.* Bioconjugate chemistry, 2016. **27**(3): p. 849-853.

195. Yihunie, W., G. Nibret, and Y. Aschale, *Recent Advances in Messenger Ribonucleic Acid (mRNA) Vaccines and Their Delivery Systems: A Review.* Clinical Pharmacology: Advances and Applications, 2023: p. 77-98.

196. Coustasse, A., C. Kimble, and K. Maxik, *COVID-19 and vaccine hesitancy: a challenge the United States must overcome.* The Journal of ambulatory care management, 2021. **44**(1): p. 71-75.

197. Ozonoff, A., E. Nanishi, and O. Levy, *Bell's palsy and SARS-CoV-2 vaccines.* The Lancet Infectious Diseases, 2021. **21**(4): p. 450-452.

198. Seneff, S. and G. Nigh, *Worse than the disease? Reviewing some possible unintended consequences of the mRNA vaccines against COVID-19.* International Journal of Vaccine Theory, Practice, and Research, 2021. **2**(1): p. 38-79.

199. Assefi, M., et al., *A state-of-the-art review on solid lipid nanoparticles as a nanovaccines delivery system.* Journal of Drug Delivery Science and Technology, 2023: p. 104623.
